# Immediate versus delayed induction of labour in hypertensive disorders of pregnancy: a systematic review and meta-analysis

**DOI:** 10.1186/s12884-020-03407-8

**Published:** 2020-11-26

**Authors:** Jia Li, Xuecheng Shao, Shurong Song, Qian Liang, Yang Liu, Xiaojin Qi

**Affiliations:** 1 Department of Obstetrics, The Third Central Hospital of Tianjin, 83 Jintang Road, Hedong District, Tianjin, 300170 China; 2 Tianjin Key Laboratory of Extracorporeal Life Support for Critical Diseases, Tianjin, China; 3 Artificial Cell Engineering Technology Research Center, Tianjin, China; 4 Tianjin Institute of Hepatobiliary Disease, Tianjin, China

**Keywords:** Delayed induction, Immediate induction, Hypertensive disorder of pregnancy, Preeclampsia

## Abstract

**Background:**

Mothers with hypertensive disorder of pregnancy can be managed with either immediate or delayed induction of labour with expectant monitoring of both mother and baby. There are risks and benefits associated with both the type of interventions. Hence, this review was conducted to compare outcomes of immediate and delayed induction of labour among women with hypertensive disorder of pregnancy based on disease severity and gestational age.

**Methods:**

We conducted systematic searches in various databases including Medline, Cochrane Controlled Register of Trials (CENTRAL), Scopus, and Embase from inception until October 2019.Cochrane risk of bias tool was used to assess the quality of published trials. A meta-analysis was performed with random-effects model and reported pooled Risk ratios (RR) with 95% confidence intervals (CIs).

**Results:**

Fourteen randomized controlled trials with 4244 participants were included. Majority of the studies had low or unclear bias risks. Amongst late onset mild pre-eclampsia patients, the risk of renal failure was significantly lower with immediate induction of labour (pooled RR: 0.36; 95%CI: 0.14 to 0.92). In severe pre-eclampsia patients, immediate induction of labour significantly reduced the risk of having small-for-gestational age babies compared to delayed induction of labour (pooled RR: 0.49; 95%CI: 0.29–0.84).Delayed induction was found to significantly reduce the risk of neonatal respiratory distress syndrome risk among late onset mild pre-eclampsia patients (pooled RR: 2.15; 95%CI: 1.14 to 4.06) None of the other outcomes demonstrated statistically significant difference between the two interventions.

**Conclusion:**

Delayed induction of labour with expectant monitoring may not be inferior to immediate induction of labour in terms of neonatal and maternal outcomes. Expectant approach of management for late onset mild pre-eclampsia patients may be associated with decreased risk of neonatal respiratory distress syndrome, while immediate induction of labour among severe pre-eclampsia patients is associated with reduced risk of small-for-gestational age babies and among mild pre-eclampsia patients, it is associated with reduced risk of severe renal impairment.

**Supplementary Information:**

The online version contains supplementary material available at 10.1186/s12884-020-03407-8.

## Background

Hypertensive disorders during pregnancy are seen in approximately 3–10% of all pregnant women and can lead to serious maternal as well as neonatal morbidity and mortality [1–3].Globally, around 80–120 women die every day because of hypertensive complication during pregnancy [4].Mothers affected by the disorder are at higher risk of developing eclampsia with seizures, renal failure, liver failure, difficulty in breathing and HELLP syndrome (Haemolysis, elevated liver enzymes and low platelet count) [5–8].

Immediate delivery of the placenta remains the only definitive management for this condition. However, delivery of preterm baby is associated with increased perinatal mortality and other short and long-term perinatal complications like respiratory distress syndrome (RDS), neonatal seizures, intracerebral haemorrhage etc. [9–13]. Another area of concern is that women with immediate induction of labour have higher caesarean section rates [14].

Alternatively, the disorder can be managed by expectant monitoring of mother and baby and delaying the labour. Expectant management consists of frequent monitoring of blood pressure, maternal symptoms (like headache, abdominal pain, blurring of vision, decrease in foetal movements, vaginal bleeding), liver & renal function test and complete blood count depending on the severity of the disease. Indications for delivery of these women include poor blood pressure control despite treatment, developing eclamptic maternal symptoms like headache, visual disturbances, epigastric pain, nausea and vomiting, unfavourable blood tests result and decrease in foetal movements. However, this line of management is associated with increased maternal morbidity and mortality wherein the disease may progress to pre-eclampsia or eclampsia, HELLP syndrome or abruptio placenta [15, 16].

In addition, maternal and neonatal outcomes with wither modes of intervention may vary depending on the severity of hypertension and gestational age [17–19]. American College of Obstericians and Gynecologists (ACOG) taskforce bulletin has indicated that a woman with pre-eclampsia should be delivered at 37 weeks of gestation [19]. Though, preterm delivery is considered as an option for severe pre-eclampsia, patients should be carefully evaluated for the adverse neonatal outcomes associated with the immediate induction of labour. Also, controversy exists on the benefits of having an elective delivery over delayed induction before34 weeks of gestation. ACOG taskforce bulletin has stated that “continued pregnancy can be undertaken at facilities only if there are adequate intensive care facilities for both mother and neonate at less than 34 weeks of gestation”. It does not provide further instructions on to whether an elective delivery needs to be performed if complications like oliguria or anuria, swelling of feet, pulmonary edema and cerebral or visual disturbances occurs [19]. Despite these recommendations, several clinicians consider that delayed induction of labour as pre-eclampsia intervention after the 34 weeks of gestation will promote a better outcome for both mothers and neonates. However, evidences supporting the management criteria are very limited.

As both the immediate and delayed induction have their own advantages and disadvantages, there is a need for high-level evidence to assess which intervention results in better outcomes for hypertensive disorder during pregnancy. Hence, the purpose of this meta-analysis is to compare maternal and neonatal outcomes following immediate induction of labour vs delayed induction for hypertensive disorder of pregnancy based on severity and gestational age.

## Methods

### Inclusion criteria

We included only randomized controlled trials (RCTs) for the current review. The inclusion criteria were as follows: studies on women with hypertensive disorder of pregnancy irrespective of disease severity and gestational age; studies comparing immediate induction of labour and delayed labour with expectant monitoring of mother and baby; studies outcomes including any of the following.

#### Maternal outcomes

Maternal mortality (death during pregnancy or up to 42 days after delivery), maternal morbidity (eclampsia, renal failure, HELLP syndrome, thromboembolic disease, postpartum haemorrhage, caesarean section rate, placental abruption).

#### Foetal/neonatal outcomes

Stillbirth rate (death of foetus at or after 28 weeks of gestation), perinatal mortality (death of foetus after 28 weeks of gestation till 7 days after delivery), neonatal mortality (death within 28 days of birth), neonatal morbidity (respiratory distress syndrome, neonatal seizures, small for gestational age, neonatal intensive care unit admission, necrotizing enterocolitis, intraventricular haemorrhage).

Non-randomised studies, single arm studies, abstracts, review articles were excluded.

### Search strategy

Relevant articles for this study were identified by searching Medline, Embase, Cochrane Central Register of Controlled Trials (CENTRAL), Scopus, ScienceDirect and Google scholar databases and search engines for studies conducted from the inception (January 1964) to October 2019. Only papers published in English were included. Trial registries such as WHO International Clinical Trials Registry Platform (ICTRP) and ClinicalTrials.gov were also searched The following medical subject headings (MeSH) were used in combination with the free text terms: “pregnancy induced hypertension” OR “hypertensive disorder of pregnancy” OR “gestational hypertension” OR “pre-eclampsia” OR “hypertension” AND (“immediate delivery” OR “immediate induction of labour” OR “induction of labour”) AND (“expectant management” OR “delayed induction of labour”) AND “pregnancy” OR “pregnant women”, with the limits to ‘human’ subjects and study design ‘randomized controlled trial’. .

All relevant studies were analyzed separately by two reviewers based on the inclusion criteria listed above. The analysis was done first at the title and abstract level and then at the full-text level. Any disagreement was resolved by discussion with a third reviewer. Additionally, reference list of full-text articles was searched for any missed-out studies. The Preferred Reporting Items for Systematic Review and Meta-Analysis (PRISMA) check list was used for reporting the current review [17].

### Data collection

The data extracted from the included studies contained all the details necessary for quality of study assessment, including: title and authors, study design and setting, participants, sample size, inclusion and exclusion criteria, characteristics of intervention and comparison groups, duration of follow up, primary and secondary study outcomes. Corresponding authors were contacted by email for missing data or when clarification or additional information was required for the methodological assessment of the included studies.

Outcome data were independently extracted by primary and secondary authors. In case of studies reporting multiple arms in a single trial, only the relevant arms were included. Collected data were transferred into the statistical software RevMan (ver 5.3) by the first author, and double checked by the third author.

### Risk of bias assessment

The risk of bias for included studies was independently assessed by two authors using Cochrane risk of bias tool for RCTs [18]. The risk of bias was assessed by: generation of random sequence, allocation concealment, blinding of the participants and outcome assessment, incomplete outcome data and selective outcome reporting.

Risk of bias was graded based on the scoring of above-mentioned domains; Grading was done as low, high or unclear based on the adequacy of information and satisfaction of the criteria.

### Statistical analysis

Meta-analysis was performed using RevMan 5.3 (Copenhagen: The Nordic Cochrane Centre, The Cochrane Collaboration, 2014). The pooled effect for the dichotomous outcomes was estimated by retrieving number of events and number of participants for each study group to calculate Relative Risk- RR. Meta-analysis was performed separately for studies with mild to moderate pre-eclampsia and severe pre-eclampsia as defined by the individual studies. Further subgroup analysis under each of these domains was carried out based on the gestational age of onset (i.e. early or late onset). Random-effects model with inverse variance was used. In case of missing data, the corresponding author of the included trial was contacted. Alternatively, imputation methods were used to fill in the missing data. Heterogeneity and inconsistencies among the included studies were assessed by chi square test and I^2^ statistics respectively. Results were graphically represented by forest plot. Selected studies were assessed for the reporting bias was assessed by comparing list of outcomes in the full study protocol with that of the published trial, where available.

## Results

### Search results

**Fig. 1:**
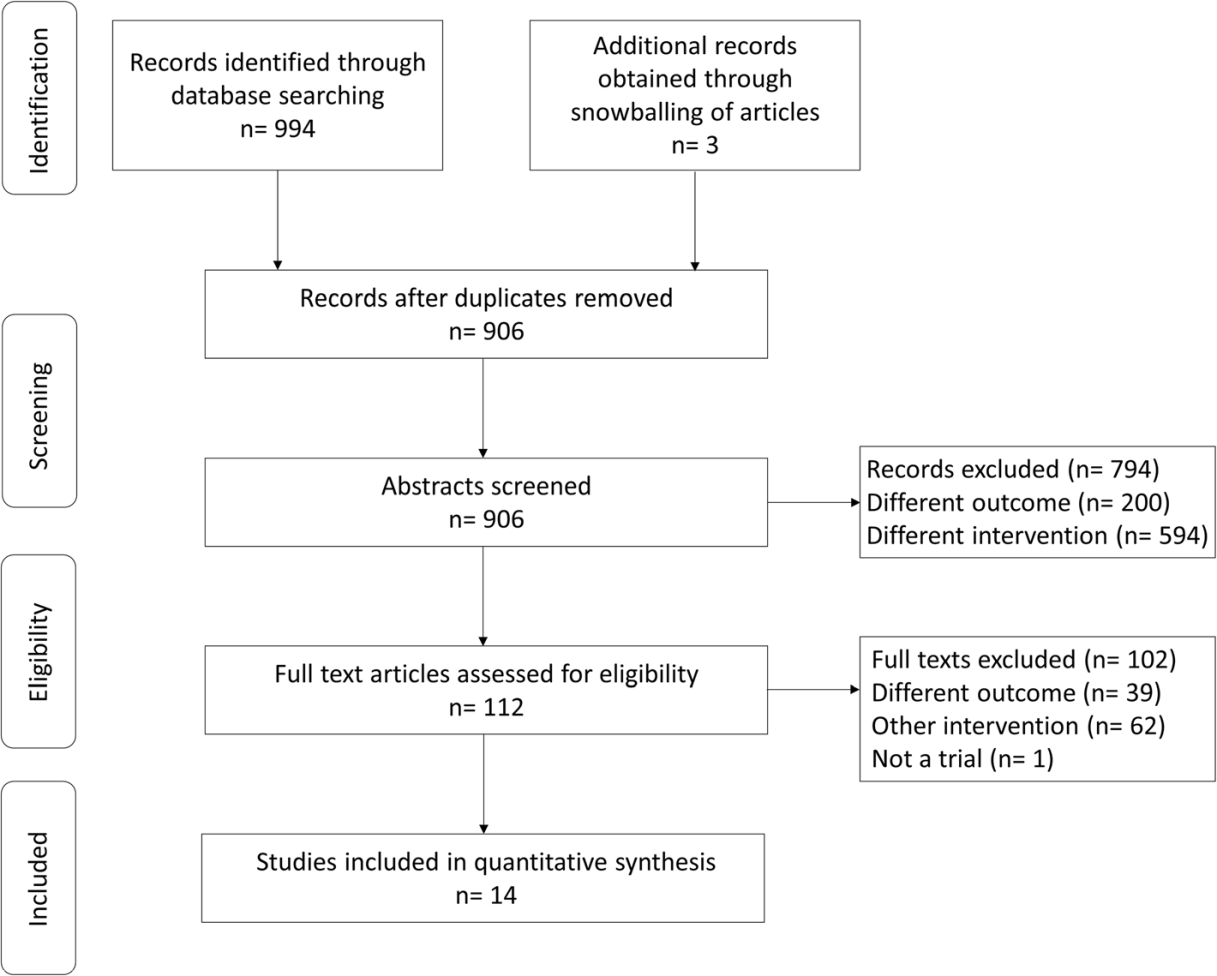
PRISMA flow chart showing the selection of studies for the current review (*n* = 14)

In total, 994 citations were identified, of which 409were retrieved from Medline, 312 from Scopus, 209 from Embase, 55 from CENTRAL, 6 from ClinicalTrials.gov and 3 from WHO ICTRP. One hundred twelve relevant studies were screened by their full texts. Bibliographies of these articles were reviewed and 3additional studies were identified. Finally, 14 studies with 4244 participants satisfying the inclusion criteria were included **(**Fig. 1**)** [15, 16, 20–33].

### Characteristics of included studies

Characteristics of the included studies are described in Table 1. All the included studies were RCTs. Most the studies (6 out of 14) were conducted in Europe. In total, 2132 participants in the immediate induction arm were compared with 2112 participants in the delayed induction arm. Total sample size of the studies varied from 30 to 946 while sample size in immediate induction arm varied from 15 to 471 and in delayed induction arm varied from 15 to 475. The definitions used for the diagnosis of gestational hypertension, pre-eclampsia and eclampsia are more or less similar across the studies included. However, there is a difference in the inclusion criteria based on the gestational age of onset and severity of the condition. Seven studies [15, 22–24, 27–29] were conducted among late onset pre-eclampsia and rest of the studies [16, 25, 26, 30–33] among early onset pre-eclampsia. Eight studies were conducted on mild to moderate pre-eclampsia patients [15, 16, 22, 23, 26–29].Six studies were conducted on severe pre-eclampsia patients [24, 25, 30–33].

**Table 1:** Characteristics of the included studies, *N* = 14

S.No	Author and year	Country	Study Design	Inclusion criteria	Sample size in early induction arm	Sample size in delayed induction arm	Gestational age of onset	Severity of pre-eclampsia	Intervention
1.	Bhageerathy et al. 2016 [22]	India	Randomized Controlled Trial	All women with a singleton pregnancy, aged 18 to 35 years, with cephalic presentation at 37 to 40 weeks of gestation, with mild gestational hypertension. Systolic BP between 140 and 159 mm of Hg and a diastolic BP between 90 and 100 mm of Hg (Korotkoff Phase V) repeated after 4 h were recruited	49	51	37–39.5 weeks (late onset)	Mild	**Experimental intervention:** For those in the immediate induction arm (group 1), a vaginal examination was done to assess the Bishop’s score. If the score was 6 or more, artificial rupture of membranes with or without oxytocin augmentation was done within 12 h of randomization. If the score was less than 6, cervical ripening was done with PGE1 (25 microgram 6th hourly for 2 doses) as is the routine for induction of labour in our hospital. **Comparison/control intervention:** For those allocated to the conservative management arm (group 2), pregnancy induced hypertension (PIH) work up which included platelet count, serum creatinine, serum transaminases (SGOT, SGPT), lactate dehydrogenase (LDH) and blood picture was done. They were advised daily home blood pressure (BP) monitoring by a local doctor or nurse who recorded it.
2.	Boers et al. 2010 [15]	Netherlands	Randomized Controlled Trial	Pregnant women between 36 + 0 and 41 + 0 weeks’ gestation who had a singleton fetus in cephalic presentation, suspected intrauterine growth restriction, and who were under specialised obstetric care were recruited.	321	329	36 weeks (Late onset)	Mild	**Experimental intervention:** Participants allocated to the induction of labour group were induced within 48 h of randomisation. If the Bishop score at randomisation was greater than 6, labour was induced with amniotomy and, if necessary, augmented with oxytocin **Comparison/Control intervention**: Participants allocated to the expectant monitoring group were monitored until the onset of spontaneous labour with daily fetal movement counts and twice weekly heart rate tracings, ultrasound examination, maternal blood pressure measurement, assessment of proteinuria, laboratory tests of liver and kidney function, and full blood count.
3.	Broekhuijsen et al. 2015 [23]	Netherlands	Randomized controlled trial	Women were eligible if they had gestational hypertension, pre-eclampsia, deteriorating pre-existing hypertension, or superimposed pre-eclampsia, and had a gestational age of 34 weeks up to and including 36 weeks. **Gestational hypertension:** diastolic blood pressure of 100 mmHg or more, on at least two occasions, 6 h apart, in women without pre-existing hypertension (defined as a blood pressure ≥ 140/90 mmHg before 20 weeks of gestation). **Pre-eclampsia:** diastolic blood pressure of 90 mmHg or more on at least two occasions, 6 h apart, combined with proteinuria, also in women without pre-existing hypertension.	352	351	34–37 weeks (Late onset)	Mild	**Experimental intervention:** planned early delivery with an induction of labour started within 24 h after randomisation **Control/Comparison intervention:** expectant monitoring until 37 weeks of GA
4.	Chappell et al. 2019 [24]	United Kingdom	Randomized controlled trial	Pregnant woman was eligible if she had a diagnosis of pre-eclampsia or superimposed pre-eclampsia (as defined by the International Society for the Study of Hypertension in Pregnancy) with a singleton or dichorionic diamniotic twin pregnancy and at least one viable fetus, was aged 18 years or older, and was able to give written informed consent. Women with any other comorbidity (including pre-existing hypertension or diabetes) or with a previous caesarean section or any foetal position were eligible.	471	475	Early onset	Severe	**Experimental intervention:** induction of labour **Control/Comparison intervention**: expectant management
5.	Duvekot et al. 2015 [25]	Netherlands	Randomized controlled Trial	Women between 28 + 0 and 34 + 0 weeks of gestation after admission for severe preeclampsia with or without HELLP syndrome	25	30	28–34 weeks (Early onset)	Severe	**Experimental intervention:** induction of labour **Control/Comparison intervention**: expectant management
6.	GRIT study group 2013 [26]	13 European countries	Randomized controlled trial	Singleton or multiple pregnancies where the responsible clinician was uncertain whether to deliver the baby immediately, the gestational age was between 24 and 36 weeks and the umbilical artery Doppler waveform had been recorded	273	274	24–36 weeks (Early onset)	Mild	**Experimental intervention:** induction of labour **Control/Comparison intervention**: expectant management
7.	Hamed et al. 2014 [16]	Saudi Arabia and Egypt	Randomized controlled Trial	Mild to moderate essential chronic hypertension without proteinuria, singleton pregnancy, and gestational age at recruitment of 24–36 weeks. Mild to moderate chronic hypertension was diagnosed if diastolic blood pressure was between 90 and 110 mmHg and/or systolic pressure was between 140 and 160 mmHg on two occasions at least 6 h apart in the first half of pregnancy, or if the patient was known to be hypertensive before pregnancy	38	38	24–36 weeks (early onset)	Mild	**Experimental intervention:** delivery at 37 completed weeks, provided that no maternal or fetal complications demanded elective preterm labour **Control/Comparison intervention:** expectant management until the spontaneous onset of labour or 41 gestational weeks
8.	Koopmans et al. 2009 [27]	Netherlands	Randomized controlled Trial	Women with a singleton pregnancy and a fetus in cephalic presentation at a gestational age of between 36 (0 days) and 41 weeks (0 days), and who had gestational hypertension or mild pre-eclampsia. **Pre-eclampsia:** diastolic BP > 90 mm on two occasions at least 6 h apart; proteinuria (two or more occurrences of protein on a dipstick, > 300 mg total protein within a 24-h urine collection, or ratio of protein to creatinine > 30 mg/mmol) **Gestational hypertension:** diastolic BP ≧ 95 mmHg, on two occasions at least 6 h apart	377	379	36 weeks (late onset)	Mild to moderate	**Experimental intervention:** induction of labour within 24 h of randomisation **Control/Comparison intervention:** expectant monitoring. They were monitored until the onset of spontaneous delivery, in hospital or outpatient setting, depending on the condition of the woman with frequent blood pressure measurements and testing of urine for protein of the mother.
9.	Owens et al. 2014 [28]	United States of America	Randomized controlled Trial	Late preterm patients with preeclampsia without severe features assigned to immediate delivery/ expectant management until 37 weeks gestation or earlier if severe features develop	94	75	34–37 weeks (late onset)	Mild	**Experimental intervention:** planned early delivery via induction of labour or caesarean delivery within 12 h of randomisation. All study participants were treated with magnesium sulphate prophylaxis intrapartum and immediately postpartum. 97 women were randomised, 3 were subsequently excluded for not meeting the inclusion criteria **Control/Comparison intervention:** inpatient expectant management, to 37 weeks’ gestation unless there was spontaneous onset of labour or rupture of membranes, suspected placental abruption, development of severe PE of fetal compromise.
10.	Majeed et al. 2014 [29]	India	Randomized controlled Trial	Pregnant women at 36 - 40 weeks’ gestation, with mild pre-eclampsia/ gestational hypertension without proteinuria **Gestational Hypertension:** systolic blood pressure ≥ 140 or diastolic blood pressure ≥ 90 mmHg for the first time during pregnancy without proteinuria. **Mild pre-eclampsia**: systolic blood pressure was 140–159 mmHg and diastolic blood pressure is 90–109 mmHg accompanied by proteinuria of > 0.3 g to < 5 g/24 h	50	50	36–40 weeks (late onset)	Mild	**Experimental intervention:** induction of labour **Control/Comparison intervention**: expectant management
11.	Mesbah EMM 2003 [30]	Egypt	Randomized controlled trial	Pregnant women with severe PE between 28 and 33 + 6 days gestation. Severe PE was defined as a BP > 180/120 mmHg on 2 occasions, 30 min apart; or a BP between 160 to 180/110 to 120 mmHg on 2 occasions, 6 h apart. All participants had > 500 mg of proteinuria on a 24 h urine collection measure	15	15	32 weeks (Early onset)	Severe	**Experimental Intervention:** Administered dexamethasone phosphate; 48 h to lapse before either an induction of labour was attempted (50 μ, vaginal misoprostol) or caesarean section after 24 h **Control/Comparison Intervention**: Administered dexamethasone phosphate then managed conservatively with bed rest, observations and nifedipine to control BP. Indications for delivery were imminent eclampsia, deteriorating renal function, spontaneous preterm labour, absent EDF or a non-reassuring CTG reaching 34 weeks
12.	Odendaal HJ et al. 1990 [32]	Africa	Randomized controlled trial	Women with severe PE at 28 to 34 weeks’ gestation. Severe PE defined in 4 ways, depending on BP, proteinuria, and symptoms. Women were either already admitted for bedrest and later met criteria, or admitted because of severe PE, and after 48 h stabilisation met entry criteria	20	18	28–34 weeks (Early onset)	Severe	**Experimental Intervention:** Delivery by induction or caesarean section depending on obstetric circumstances 48 h after betamethasone. If cervix not favourable, prostaglandin E2 tablets. If still not favourable after 24 h, caesarean section **Control/Comparison intervention**: Bed rest on high-risk obstetric ward; maternal and fetal condition monitored intensively; BP controlled with prazosin; delivery at 34 weeks unless indicated earlier
13.	Sibai BM et al. 1994 [33]	United States of America	Randomized controlled trial	Women with severe PE at 28 to 32 weeks’ gestation. Severe PE defined as a persistent elevation of BP ≥ 160/110 mmHg, proteinuria > 500 mg in 24 h, and uric acid > 5 mg/dL.	46	49	32 weeks (Early onset)	Severe	**Experimental Intervention:** Delivery by caesarean section or by induction of labour, on the basis of obstetric condition, 48 h after first dose of betamethasone **Control/Comparison intervention**: Maternal and fetal monitoring on an antenatal ward. If either condition deteriorated, or reached 34 weeks’ gestation, delivery using the ‘most appropriate method’
15.	Vigil De Gracia et al. 2013 [31]	Latin America	Randomized controlled trial	Pregnant women between 28 and 33 weeks’ gestation with severe PE, severe gestational hypertension, and super-imposed PE	133	131	32 weeks (Early onset)	Severe	**Experimental intervention:** Prompt delivery’: glucocorticoid therapy followed by delivery in 24–72 h, magnesium sulphate continued until 24 h after delivery **Control/Comparison intervention**: Treated expectantly: glucocorticoid therapy followed by delivery only for specific maternal/ fetal indications or reaching 34 weeks of gestation

### Quality of the included studies

Authors assessment of risk of bias for included studies is presented in Table 2. Most of the studies had low risk of bias with respect to the randomization process (random sequence generation and allocation concealment). All the included studies had either high or unclear risk of bias with respect to blinding of participants and outcome assessment. All the studies had low or unclear risk of bias with respect to incomplete outcome data and selective reporting of outcome except Majeed et al. [29] and Mesbah EMM [30].

**Table 2:** Risk of bias assessment for the included studies, *N* = 14

S.No	Author and year	Random sequence generation	Allocation concealment	Blinding of the participants	Blinding of outcome assessment	Incomplete outcome data	Selective reporting of outcome
1.	Bhageerathy et al. 2016 [22]	Low risk	Low risk	High risk	High risk	Low risk	Unclear risk
2.	Boers et al. 2010 [15]	Low risk	Low risk	High risk	Unclear risk	Low risk	Low risk
3.	Broekhuijsen et al. 2015 [23]	Low risk	Low risk	High risk	Unclear risk	Low risk	Low risk
4.	Chappell et al. 2019 [24]	Unclear risk	Unclear risk	High risk	High risk	Unclear risk	Unclear risk
5.	Duvekot et al. 2015 [25]	Low risk	Low risk	High risk	High risk	Low risk	Unclear risk
6.	GRIT study group 2013 [26]	Low risk	Unclear risk	High risk	Unclear risk	Unclear risk	Low risk
7.	Hamed et al. 2014 [16]	Low risk	Unclear risk	High risk	High risk	Low risk	Unclear risk
8.	Koopmans et al. 2009 [27]	Low risk	Low risk	High risk	Unclear risk	Low risk	Low risk
9.	Owens et al. 2014 [28]	Low risk	Unclear risk	High risk	Unclear risk	Unclear risk	Low risk
10.	Majeed et al. 2014 [29]	Unclear risk	Unclear risk	High risk	High risk	Unclear risk	High risk
11.	Mesbah EMM 2003 [30]	Low risk	Low risk	Unclear risk	Unclear risk	High risk	Low risk
12.	Odendaal HJ et al. 1990 [32]	Unclear risk	Unclear risk	Unclear risk	Unclear risk	Unclear risk	Unclear risk
13.	Sibai BM et al. 1994 [33]	Low risk	Low risk	Unclear risk	Unclear risk	Low risk	Low risk
14.	Vigil De Gracia et al. 2013 [31]	Low risk	Low risk	Unclear risk	Unclear risk	Low risk	Low risk

### Outcomes

All the outcome estimates based on the gestational age of onset and severity of condition is provided in the Table 3.

**Table 3:** Summary of findings among studies comparing immediate induction with delayed induction of labour

Outcome	Number of studies pooled	Events Immediate Induction of labour	Total Immediate Induction of labour	Events Delayed induction	Total Delayed induction	Pooled RR (95% CI)	I^**2**^	Reference	Figure
**Early onset severe pre-eclampsia**									
Maternal mortality	3	0	630	1	636	0.34 (0.01–8.23)	NA	[24, 25, 31]	Additional file 2: Appendix 1.1
Eclampsia	2	1	179	1	180	0.98 (0.06–15.58)	NA	[31, 33]	Additional file 2: Appendix 1.4
Severe renal impairment	3	1	199	4	198	0.32 (0.05–1.99)	0%	[31–33]	Additional file 2: Appendix 1.6
HELLP syndrome	4	7	872	18	856	0.40 (0.17–0.94)	0%	[22, 23, 27, 28]	Additional file 2: Appendix 1.7
Caesarean section	6	463	710	500	718	0.95 (0.89–1.01)	10%	[24, 25, 30–33]	Additional file 2: Appendix 1.13
Placental abruption	4	7	225	17	228	0.47 (0.20–1.12)	0%	[25, 31–33]	Additional file 2: Appendix 1.14
Stillbirths	5	1	689	2	695	0.60 (0.07–4.73)	0%	[24, 30–33]	Additional file 2: Appendix 1.16
Perinatal mortality	5	21	672	19	677	1.14 (0.64–2.02)	0%	[24, 25, 30–32]	Additional file 2: Appendix 1.19
Neonatal mortality	5	10	581	6	588	1.60 (0.66–3.88)	0%	[24, 25, 30–32]	Additional file 2: Appendix 1.22
Respiratory distress syndrome	3	81	537	56	542	1.69 (1.00–2.85)	55%	[24, 32, 33]	Additional file 2: Appendix 1.24
Small for gestational age babies	4	94	669	149	677	0.49 (0.29–0.84)	60%	[24, 30, 31, 33]	Additional file 2: Appendix 1.29
Neonatal intensive care unit admission	4	183	669	168	667	1.22 (0.95–1.56)	75%	[24, 30, 31, 33]	Additional file 2: Appendix 1.32
Intraventricular haemorrhage	1	4	137	1	138	4.03 (0.46–35.59)	NA	[33]	–
Necrotizing enterocolitis	3	9	203	3	205	2.23 (0.42–11.87)	29%	[31–33]	Additional file 2: Appendix 1.33
**Late onset mild pre-eclampsia**									
Maternal mortality	4	1	1099	0	1110	3.07 (0.13–75.19)	NA	[15, 22, 23, 27]	Additional file 2: Appendix 1.2
Eclampsia	4	1	1099	2	1110	0.76 (0.05–11.18)	34%	[15, 22, 23, 27]	Additional file 2: Appendix 1.3
Severe renal impairment	2	5	99	14	101	0.36 (0.14–0.92)	NA	[22, 29]	Additional file 2: Appendix 1.5
HELLP syndrome	2	22	179	19	180	1.15 (0.65–2.02)	0%	[31, 33]	Additional file 2: Appendix 1.8
Thromboembolic disease	3	2	1050	1	1059	1.60 (0.20–12.99)	0%	[15, 23, 27]	Additional file 2: Appendix 1.9
Postpartum haemorrhage	3	46	740	57	751	0.82 (0.56–1.19)	0%	[15, 22, 27]	Additional file 2: Appendix 1.10
Caesarean section	6	276	1243	288	1235	0.95 (0.82–1.09)	0%	[15, 22, 23, 27–29]	Additional file 2: Appendix 1.11
Stillbirths	3	1	814	5	801	0.17 (0.02–1.45)	NA	[15, 23, 26]	Additional file 2: Appendix 1.15
Perinatal mortality	4	1	1099	0	1110	3.12 (0.13–74.80)	NA	[15, 22, 23, 27]	Additional file 2: Appendix 1.18
Neonatal mortality	4	1	1099	0	1110	3.12 (0.13–74.80)	NA	[15, 22, 23, 27]	Additional file 2: Appendix 1.21
Respiratory distress syndrome	3	32	771	13	740	2.15 (1.14–4.06)	0%	[23, 27, 28]	Additional file 2: 1.23
Neonatal seizures	1	4	351	1	348	3.97 (0.45–35.30)	NA	[23]	Additional file 2: Appendix 1.26
Small for gestational age babies	4	240	841	256	834	1.19 (0.73–1.94)	45%	[15, 22, 27, 28]	Additional file 2: Appendix 1.27
Neonatal intensive care unit admission	5	68	1140	49	1119	1.28 (0.87–1.87)	10%	[15, 22, 23, 27, 28]	Additional file 2: Appendix 1.30
**Early onset mild pre-eclampsia**									
Caesarean	2	152	179	113	159	1.49 (0.68–3.23)	73%	[16, 26]	Additional file 2: Appendix 1.12
Perinatal mortality	2	27	334	22	329	1.20 (0.70–2.07)	0%	[16, 26]	Additional file 2: Appendix 1.17
Neonatal mortality	2	23	179	16	159	1.24 (0.68–2.25)	0%	[16, 26]	Additional file 2: Appendix 1.20
Neonatal seizures	1	3	141	1	121	2.57 (0.27–24.43)	NA	[16]	Additional file 2: Appendix 1.25
Small for gestational age babies	1	6	38	4	38	1.50 (0.46–4.89)	NA	[16]	Additional file 2: Appendix 1.28
Neonatal intensive care unit admission	1	12	38	3	38	4.00 (1.23–13.05)	NA	[16]	Additional file 2: Appendix 1.31
Intraventricular haemorrhage	1	34	141	16	121	1.82 (1.06–3.14)	NA	[26]	–

*NA* Not applicable

### Maternal outcomes

#### Maternal mortality

Seven studies reported data on maternal mortality. Five of those studies had zero incidence of maternal mortality in both study groups Stratification based on severity and gestational age of onset had one study each under mild [15] and severe pre-eclampsia [24] and both were conducted among late onset pre-eclampsia patients. The RR in mild pre-eclampsia group was 3.07 (95%CI: 0.13–75.19) and severe pre-eclampsia group was 0.34 (0.01–8.23). Both showing non-significant difference in terms of maternal mortality **(**Additional file 2: Appendix 1.1 & 1.2).

### Maternal morbidity

Six studies reported data on eclampsia, of which, Meta-analysis indicated no statistical significant difference in the risk of eclampsia for late onset mild pre-eclampsia [RR:0.76 (95%CI: 0.05 to 11.18); I^2^ = 34%] **(**Additional file 2: Appendix 1.3) as well as for early onset severe pre-eclampsia [RR: 0.98 (95%CI: 0.06–15.58); I^2^ = not applicable] **(**Additional file 2: Appendix 1.4). Five studies reported data on severe renal impairment. Meta-analysis indicated statistically significant reduced risk of renal failure in induction arm amongst late onset mild pre-eclampsia patients [RR: 0.36 (95%CI: 0.14 to 0.92); I^2^ = not applicable] **(**Additional file 2: Appendix 1.5) but no difference for early onset severe pre-eclampsia patients [RR: 0.32 (95%CI: 0.05–1.99); I^2^ = 0%] **(**Additional file 2: Appendix 1.6). Six studies reported data on incidence of HELLP syndrome among mothers following immediate or delayed induction of labour. Pooled analysis was insignificant for both late onset mild pre-eclampsia [RR:0.40 (95%CI: 0.17 to 11.94); I^2^ = 0%] **(**Additional file 2: Appendix 1.7) and early onset severe pre-eclampsia [RR:1.15 (95%CI: 0.65–2.02); I^2^ = 0%] (Additional file 2: Appendix 1.8).

Three studies reported data on the risk of thromboembolic disease. All the three studies were conducted among late onset mild pre-eclampsia patients. Meta-analysis indicated no statistically significant difference in risk of thromboembolic disease between the two groups [RR:1.60 (95%CI: 0.20 to 12.99); I^2^ = 0%] **(**Additional file 2: Appendix 1.9).

Three studies reported data on the risk of postpartum haemorrhage. All the three studies were conducted among late onset mild pre-eclampsia patients. Meta-analysis indicated no statistically significant difference in risk of postpartum haemorrhage between the two groups [RR:0.82 (95%CI: 0.56 to 1.19); I^2^ = 0%] **(**Additional file 2: Appendix 1.10). All the 14 studies have reported on the incidence of caesarean section among mothers in both the arms. The pooled RR for late onset mild pre-eclampsia was 0.95 (95%CI: 0.82 to 1.09, I^2^ = 0%) (Additional file 2: Appendix 1.11) and early onset mild pre-eclampsia was 2.23 (95%CI: 0.42–11.87, I^2^ = 29%). (Additional file 2: Appendix 1.12). For early onset severe pre-eclampsia, pooled RR was 0.95 (95%CI: 0.89–1.01; I^2^ = 10%) **(**Additional file 2: Appendix 1.13). There was a significant publication bias as depicted by asymmetrical funnel plot **(**Additional file 1: Supplementary Fig. 1). Four studies have reported on placental abruption and all of them were conducted among early-onset severe pre-eclampsia patients. The pooled RR was 0.47 (95%CI: 0.20–1.12; I^2^ = 0%) **(**Additional file 2: Appendix 1.14).

#### Foetal/ neonatal outcome

Eight studies have reported on the stillbirth rate in both the arms. First, among mild pre-eclampsia studies, stillbirth occurred in only one study. The RR was 0.17 (95%CI: 0.02 to 1.45; I^2^ = not applicable) **(**Additional file 2: Appendix 1.15). For severe pre-eclampsia, the pooled RR was 0.60 (95%CI: 0.07–4.73; I^2^ = 0%) (Additional file 2: Appendix 1.16). Eleven studies have reported on the perinatal mortality in both the arms. The pooled RR for early mild pre-eclampsia patients was 1.20 (95%CI: 0.70 to 2.07; I^2^ = 0%) **(**Additional file 2: Appendix 1.17). Four studies have reported perinatal deaths on late onset mild patients, out of which only study reported perinatal deaths with RRof3.12 (95%CI: 0.13 to 74.80; I^2^ = NA) **(**Additional file 2: Appendix 1.18). Five studies reported among early onset severe patients with pooled RR of 3.12 (95%CI: 0.13–74.80, I^2^ = 0%) **(**Additional file 2: Appendix 1.19). Eleven studies have reported on the neonatal mortality in both the arms. Out of these, six studies were conducted among mild pre-eclampsia patients (2 studies among early onset and 4 among late onset patients). The pooled RR for early onset mild pre-eclampsia was 1.24 (95%CI: 0.68 to 2.25; I^2^ = 0%) **(**Additional file 2: Appendix 1.20) and for late onset patients was 3.12 (95%CI: 0.13–74.80, I^2^ = 0%) **(**Additional file 2: Appendix 1.21). Five studies were conducted among early severe pre-eclampsia patients. The pooled RR was 1.60 (95%CI: 0.66 to 3.88; I^2^ = 0%) **(**Additional file 2: Appendix 1.22).

Six studies have reported on the risk of respiratory distress syndrome among neonates in both the arms. Out of these, three studies were conducted among late onset mild pre-eclampsia patients. The pooled RR was 2.15 (95%CI: 1.14 to 4.06; I^2^ = 0%) **(**Additional file 2: Appendix 1.23). Three other studies were conducted among early onset severe pre-eclampsia patients. The pooled RR was 1.69 (95%CI: 1.00–2.85; I^2^ = 55%) **(**Additional file 2: Appendix 1.24). Two studies have reported on the risk of seizures among neonates in both the arms. Both the studies were conducted among mild pre-eclampsia patients. However, one study was conducted among early onset patients with RR of 2.57 (95%CI: 0.27–24.43) and one among late onset patients with RR of 3.97 (95%CI: 0.45–35.30) **(**Additional file 2: Appendix 1.25 & 1.26). Nine studies have reported on the risk of small for gestational age (SGA) babies among neonates in both the arms. Five studies were conducted among mild pre-eclampsia patients (one in early onset and four in late onset patients). The pooled RR of late onset patients was 1.19 (95%CI: 0.73 to 1.94; I^2^ = 45%) **(**Additional file 2: Appendix 1.27) and RR of early onset patients was 1.50 (95%CI: 0.46–4.89, I^2^ = NA) **(**Additional file 2: Appendix 1.28). Four studies were conducted among early onset severe pre-eclampsia patients. The pooled RR was 0.49 (95%CI: 0.29–0.84; I^2^ = 60%) **(**Additional file 2: Appendix 1.29).

Ten studies have reported on the neonatal intensive care unit (NICU) admission in both the arms. Six studies were conducted among mild pre-eclampsia patients (one early onset and five late onset pre-eclampsia patients). The pooled RR for late onset patients was 1.28 (95%CI: 0.87 to 1.87; I^2^ = 10%) **(**Additional file 2: Appendix 1.30) and RR for early onset patients was 4.00 (95%CI: 1.23–13.05, I^2^ = NA) **(**Additional file 2: Appendix 1.31). Four studies were conducted among early onset severe pre-eclampsia patients. The pooled RR was 1.22 (95%CI: 0.95–1.56; I^2^ = 75%) (Additional file 2: Appendix 1.32). Two studies have reported on intraventricular haemorrhage in both the arms (one study among early onset severe pre-eclampsia patients and other in late onset mild pre-eclampsia patients). The RR reported in early onset severe patients were 4.03 (95%CI: 0.46–35.59; I^2^ = Not applicable) and early onset mild patients were 1.82 (1.06–3.14; I^2^ = Not applicable). Four studies have reported on necrotizing enterocolitis, out of which three were conducted among early onset severe patients and only one among early onset mild patients. The pooled RR for early onset severe patients were 2.23 (95%CI: 0.42–11.87; I^2^ = 29%) **(**Additional file 2: Appendix 1.33).

## Discussion

Mothers with hypertensive disorder of pregnancy can be managed with either immediate induction of labour or delayed induction with expectant monitoring of both mother and baby. There are risks and benefits associated with both the type of interventions. Hence, it is important to analyse literature so as to which intervention is associated with better maternal and foetal outcomes.

In all, we identified 14 studies with 4244 participants for our analysis. Majority of the studies were conducted in countries of European region and American region. Most of the included studies had low risk of bias with respect to all the domains except blinding process domains. We did not find any substantial heterogeneity for most of the outcomes in the studies except studies reporting caesarean section rate in both the arms.

Only three outcomes (one maternal and two neonatal outcomes) showed a statistically significant difference between the two interventions. Immediate induction of labour was beneficial to mothers as it had reduced risk of severe renal impairment among late onset mild pre-eclampsia patients. It was also beneficial in neonatal outcome in terms of SGA babies among severe pre-eclampsia patients. Our analysis also demonstrated that delayed induction significantly reduces the risk of neonatal respiratory distress syndrome among late onset mild pre-eclampsia patients. Our results are similar to the previous Cochrane review conducted by Churchill D et al. in 2018 [34]. In their study too, immediate induction of labour was associated with reduced risk of SGA babies and the risk of neonatal respiratory distress syndrome was found to be lower with delayed induction and expectant management. In our results, no statistically significant difference was noted in any of the other outcomes as the confidence interval crossed the null value in all our remaining analyses. This indicates that evidence on the superiority of one intervention over the other for managing women with hypertensive disorder of pregnancy is limited.

The ACOG guidelines classify mild pre-eclampsia as systolic blood pressure of 140-149 mmHg and/or diastolic pressure of 90-99 mmHg, in women with a previously normal blood pressure with proteinuria of ≥300 mg/24-h urine collection. On the other hand, severe pre-eclampsia is classified as systolic blood pressure of > 160 mmHg and/or diastolic pressure of > 110 mmHg with severe proteinuria (2-5 g/24-h urine collection) [19]. It is important to note that majority of the include studies in our analysis were conducted on early onset severe pre-eclampsia and late onset mild pre-eclampsia. The number of studies pooled for a meta-analysis on maternal and neonatal outcomes for early onset mild pre-eclampsia were limited to just two. While our analysis did not find any difference between the two interventions for incidence of caesarean section, perinatal mortality and neonatal mortality with pooled analysis of the two studies, lack of data significantly limits the ability of our review to derive strong conclusions for this sub-group. In view of this, clinicians should carefully weigh the risk vs benefits of each intervention on a case-to-case basis to achieve optimal maternal and fetal outcomes for patients with early mild pre-eclampsia.

Three other similar reviews have been conducted on this topic, a meta-analysis of RCTs by Cluver C et al. (2017), Wang Y et al. (2017) and individual participant data meta-analysis by Bernandes et al. (2019) have also reported almost similar findings compared to our review [35–37]. However, the number of studies included in these previous reviews have been limited to only 4–5 trials. In comparison, the current review has analysed 14 studies and comprehensively reviewed the maternal and neonatal morbidity & mortality outcomes with immediate induction and delayed induction of labour. We believe, the updated evidence shall help healthcare personnel in appropriately choosing the timing of induction of labour for the patients with hypertensive disorder of pregnancy.

The major strengths of our study include the comprehensive search of literature and the broad search strategy resulting in inclusion of all studies published on the topic to date. We only included RCTs into our review which enables us to infer causal associations between the intervention and outcomes. Several maternal and neonatal outcomes were analysed in our study thereby providing a comprehensive comparison of the two intervention modalities. The level of inter-study heterogeneity among majority of the maternal or neonatal outcomes was low. Outcomes were stratified based on the disease severity and gestational age of onset to take into account the influence of such confounding factors on the overall outcome.

We are also aware of the limitations of our review. We could not assess the possibility of publication bias for majority outcomes due to the limited number of included studies. Finally, most of the studies included in our review were conducted in European region, which may limit the generalizability of our findings to other geographical areas.

## Conclusion

To summarize, delayed induction of labour with expectant monitoring may not be inferior to immediate induction of labour in terms of maternal and neonatal outcomes. Expectant approach of management for late onset mild pre-eclampsia patients may be associated with decreased risk of neonatal respiratory distress syndrome. Immediate induction of labour among severe pre-eclampsia patients is associated with reduced risk of SGA babies and among mild pre-eclampsia patients, it may be associated with reduced risk of severe renal impairment. Evidence on early onset mild pre-eclampsia was limited to draw strong conclusions.

## Supplementary Information

**Additional file 1:** **Figure S1.** Funnel plot checking for publication bias (*n* = 14).

**Additional file 2:** **Appendix 1.1.** Forest plot showing the difference in maternal mortality between immediate and delayed induction of labour among early onset severe pre-eclampsia patients. **Appendix 1.2.** Forest plot showing the difference in maternal mortality between immediate and delayed induction of labour among late onset mild pre-eclampsia patients. **Appendix 1.3.** Forest plot showing the difference in eclampsia between immediate and delayed induction of labour among late onset mild pre-eclampsia patients. **Appendix 1.4.** Forest plot showing the difference in eclampsia between immediate and delayed induction of labour among early onset severe pre-eclampsia patients. **Appendix 1.5.** Forest plot showing the difference in renal failure between immediate and delayed induction of labour among late onset mild pre-eclampsia patients. **Appendix 1.6.** Forest plot showing the difference in renal failure between immediate and delayed induction of labour among early onset severe pre-eclampsia patients. **Appendix 1.7.** Forest plot showing the difference in HELLP syndrome between immediate and delayed induction of labour among early onset severe pre-eclampsia patients. **Appendix 1.8.** Forest plot showing the difference in HELLP syndrome between immediate and delayed induction of labour among late onset mild pre-eclampsia patients. **Appendix 1.9.** Forest plot showing the difference in thromboembolic disease between immediate and delayed induction of labour among late onset mild pre-eclampsia patients. **Appendix 1.10.** Forest plot showing the difference in postpartum haemorrhage between immediate and delayed induction of labour among late onset mild pre-eclampsia patients. **Appendix 1.11.** Forest plot showing the difference in caesarean section between immediate and delayed induction of labour among late onset mild pre-eclampsia patients. **Appendix 1.12.** Forest plot showing the difference in caesarean section between immediate and delayed induction of labour among early onset mild pre-eclampsia patients. **Appendix 1.13.** Forest plot showing the difference in caesarean section between immediate and delayed induction of labour among early onset severe pre-eclampsia patients. **Appendix 1.14.** Forest plot showing the difference in placental abruption between immediate and delayed induction of labour among early onset severe pre-eclampsia patients. **Appendix 1.15.** Forest plot showing the difference in stillbirth between immediate and delayed induction of labour among late onset mild pre-eclampsia patients. **Appendix 1.16.** Forest plot showing the difference in stillbirth between immediate and delayed induction of labour among early onset severe pre-eclampsia patients. **Appendix 1.17.** Forest plot showing the difference in perinatal mortality between immediate and delayed induction of labour among early onset mild pre-eclampsia patients. **Appendix 1.18.** Forest plot showing the difference in perinatal mortality between immediate and delayed induction of labour among late onset mild pre-eclampsia patients. **Appendix 1.19.** Forest plot showing the difference in perinatal mortality between immediate and delayed induction of labour among early onset severe pre-eclampsia patients. **Appendix 1.20.** Forest plot showing the difference in neonatal mortality between immediate and delayed induction of labour among early onset mild pre-eclampsia patients. **Appendix 1.21.** Forest plot showing the difference in neonatal mortality between immediate and delayed induction of labour among late onset mild pre-eclampsia patients. **Appendix 1.22.** Forest plot showing the difference in neonatal mortality between immediate and delayed induction of labour among early onset severe pre-eclampsia patients. **Appendix 1.23.** Forest plot showing the difference in neonatal respiratory distress syndrome between immediate and delayed induction of labour among late onset mild pre-eclampsia patients. **Appendix 1.24.** Forest plot showing the difference in neonatal respiratory distress syndrome between immediate and delayed induction of labour among early onset severe pre-eclampsia patients. **Appendix 1.25.** Forest plot showing the difference in neonatal seizures between immediate and delayed induction of labour among early onset mild pre-eclampsia patients. **Appendix 1.26.** Forest plot showing the difference in neonatal seizures between immediate and delayed induction of labour among early onset severe pre-eclampsia patients. **Appendix 1.27.** Forest plot showing the difference in small for gestational age babies between immediate and delayed induction of labour among late onset mild pre-eclampsia patients. **Appendix 1.28.** Forest plot showing the difference in small for gestational age babies between immediate and delayed induction of labour among early onset mild pre-eclampsia patients. **Appendix 1.29.** Forest plot showing the difference in small for gestational age babies between immediate and delayed induction of labour among early onset severe pre-eclampsia patients. **Appendix 1.30.** Forest plot showing the difference in neonatal intensive care unit admission rate between immediate and delayed induction of labour among late onset mild pre-eclampsia patients. **Appendix 1.31.** Forest plot showing the difference in neonatal intensive care unit admission rate between immediate and delayed induction of labour among early onset mild pre-eclampsia patients. **Appendix 1.32.** Forest plot showing the difference in neonatal intensive care unit admission rate between immediate and delayed induction of labour among early onset severe pre-eclampsia patients. **Appendix 1.33.** Forest plot showing the difference in necrotizing enterocolitis between immediate and delayed induction of labour among early onset severe pre-eclampsia patients

## Data Availability

The datasets used and/or analyzed during the current study are available from the corresponding author on reasonable request.

## Abbreviations

CENTRAL: Cochrane Controlled Register of Trials
RR: Risk ratios
CIs: Confidence intervals
HELLP: Haemolysis, elevated liver enzymes and low platelet
RDS: Respiratory distress syndrome
ICTRP: International Clinical Trials Registry Platform
MeSH: Medical subject heading
PRISMA: Preferred Reporting Items for Systematic Review and Meta-Analysis
RCT: Randomized Controlled Trials
NICU: Neonatal intensive care unit
