# Supplementary figures and images for: Immediate versus delayed induction of labour in hypertensive disorders of pregnancy: a systematic review and meta-analysis
Source: BMC Pregnancy Childbirth. 2020 Nov 26;20:735. doi: 10.1186/s12884-020-03407-8 (PMC7690081; doi:10.1186/s12884-020-03407-8)

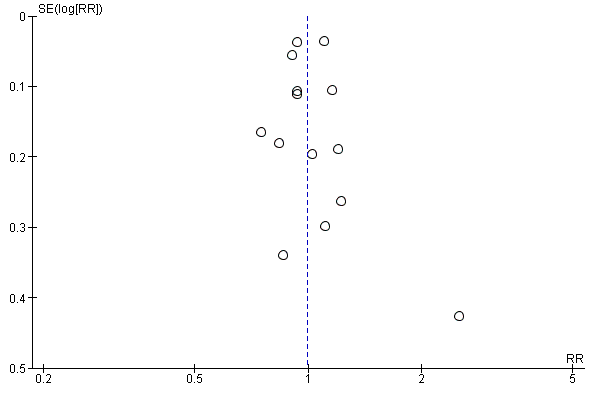

Supplement: Supplementary file 1 — Additional file 1: Figure S1. Funnel plot checking for publication bias (n = 14). [file 12884_2020_3407_MOESM1_ESM.tif]

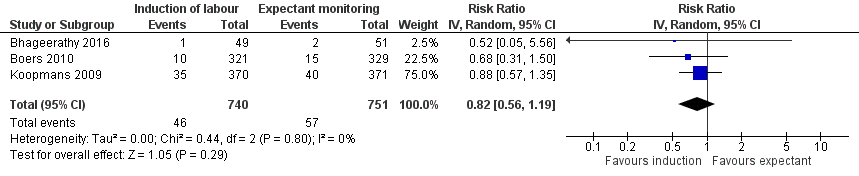

Supplement: Supplementary file 2 — Additional file 2: Appendix 1.1. Forest plot showing the difference in maternal mortality between immediate and delayed induction of labour among early onset severe pre-eclampsia patients. Appendix 1.2. Forest plot showing the difference in maternal mortality between immediate and delayed induction of labour among late onset mild pre-eclampsia patients. Appendix 1.3. Forest plot showing the difference in eclampsia between immediate and delayed induction of labour among late onset mild pre-eclampsia patients. Appendix 1.4. Forest plot showing the difference in eclampsia between immediate and delayed induction of labour among early onset severe pre-eclampsia patients. Appendix 1.5. Forest plot showing the difference in renal failure between immediate and delayed induction of labour among late onset mild pre-eclampsia patients. Appendix 1.6. Forest plot showing the difference in renal failure between immediate and delayed induction of labour among early onset severe pre-eclampsia patients. Appendix 1.7. Forest plot showing the difference in HELLP syndrome between immediate and delayed induction of labour among early onset severe pre-eclampsia patients. Appendix 1.8. Forest plot showing the difference in HELLP syndrome between immediate and delayed induction of labour among late onset mild pre-eclampsia patients. Appendix 1.9. Forest plot showing the difference in thromboembolic disease between immediate and delayed induction of labour among late onset mild pre-eclampsia patients. Appendix 1.10. Forest plot showing the difference in postpartum haemorrhage between immediate and delayed induction of labour among late onset mild pre-eclampsia patients. Appendix 1.11. Forest plot showing the difference in caesarean section between immediate and delayed induction of labour among late onset mild pre-eclampsia patients. Appendix 1.12. Forest plot showing the difference in caesarean section between immediate and delayed induction of labour among early onset mild pre-eclampsia p [file 12884_2020_3407_MOESM2_ESM.zip › Appendix 1.10R5.png]

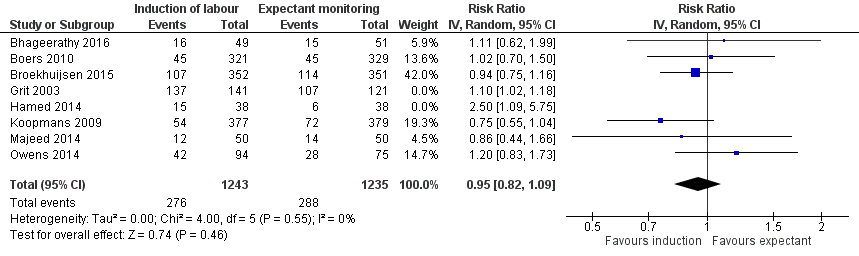

Supplement: Supplementary file 2 — Additional file 2: Appendix 1.1. Forest plot showing the difference in maternal mortality between immediate and delayed induction of labour among early onset severe pre-eclampsia patients. Appendix 1.2. Forest plot showing the difference in maternal mortality between immediate and delayed induction of labour among late onset mild pre-eclampsia patients. Appendix 1.3. Forest plot showing the difference in eclampsia between immediate and delayed induction of labour among late onset mild pre-eclampsia patients. Appendix 1.4. Forest plot showing the difference in eclampsia between immediate and delayed induction of labour among early onset severe pre-eclampsia patients. Appendix 1.5. Forest plot showing the difference in renal failure between immediate and delayed induction of labour among late onset mild pre-eclampsia patients. Appendix 1.6. Forest plot showing the difference in renal failure between immediate and delayed induction of labour among early onset severe pre-eclampsia patients. Appendix 1.7. Forest plot showing the difference in HELLP syndrome between immediate and delayed induction of labour among early onset severe pre-eclampsia patients. Appendix 1.8. Forest plot showing the difference in HELLP syndrome between immediate and delayed induction of labour among late onset mild pre-eclampsia patients. Appendix 1.9. Forest plot showing the difference in thromboembolic disease between immediate and delayed induction of labour among late onset mild pre-eclampsia patients. Appendix 1.10. Forest plot showing the difference in postpartum haemorrhage between immediate and delayed induction of labour among late onset mild pre-eclampsia patients. Appendix 1.11. Forest plot showing the difference in caesarean section between immediate and delayed induction of labour among late onset mild pre-eclampsia patients. Appendix 1.12. Forest plot showing the difference in caesarean section between immediate and delayed induction of labour among early onset mild pre-eclampsia p [file 12884_2020_3407_MOESM2_ESM.zip › Appendix 1.11R5.png]

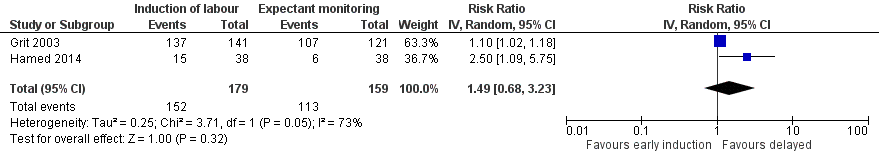

Supplement: Supplementary file 2 — Additional file 2: Appendix 1.1. Forest plot showing the difference in maternal mortality between immediate and delayed induction of labour among early onset severe pre-eclampsia patients. Appendix 1.2. Forest plot showing the difference in maternal mortality between immediate and delayed induction of labour among late onset mild pre-eclampsia patients. Appendix 1.3. Forest plot showing the difference in eclampsia between immediate and delayed induction of labour among late onset mild pre-eclampsia patients. Appendix 1.4. Forest plot showing the difference in eclampsia between immediate and delayed induction of labour among early onset severe pre-eclampsia patients. Appendix 1.5. Forest plot showing the difference in renal failure between immediate and delayed induction of labour among late onset mild pre-eclampsia patients. Appendix 1.6. Forest plot showing the difference in renal failure between immediate and delayed induction of labour among early onset severe pre-eclampsia patients. Appendix 1.7. Forest plot showing the difference in HELLP syndrome between immediate and delayed induction of labour among early onset severe pre-eclampsia patients. Appendix 1.8. Forest plot showing the difference in HELLP syndrome between immediate and delayed induction of labour among late onset mild pre-eclampsia patients. Appendix 1.9. Forest plot showing the difference in thromboembolic disease between immediate and delayed induction of labour among late onset mild pre-eclampsia patients. Appendix 1.10. Forest plot showing the difference in postpartum haemorrhage between immediate and delayed induction of labour among late onset mild pre-eclampsia patients. Appendix 1.11. Forest plot showing the difference in caesarean section between immediate and delayed induction of labour among late onset mild pre-eclampsia patients. Appendix 1.12. Forest plot showing the difference in caesarean section between immediate and delayed induction of labour among early onset mild pre-eclampsia p [file 12884_2020_3407_MOESM2_ESM.zip › Appendix 1.12R5.png]

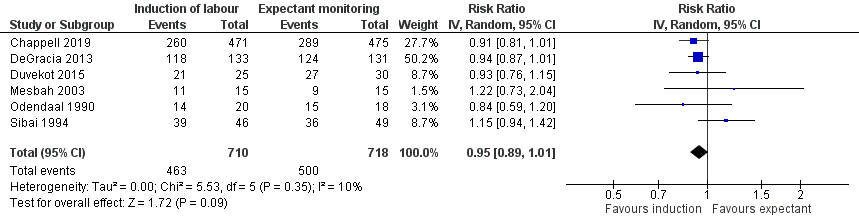

Supplement: Supplementary file 2 — Additional file 2: Appendix 1.1. Forest plot showing the difference in maternal mortality between immediate and delayed induction of labour among early onset severe pre-eclampsia patients. Appendix 1.2. Forest plot showing the difference in maternal mortality between immediate and delayed induction of labour among late onset mild pre-eclampsia patients. Appendix 1.3. Forest plot showing the difference in eclampsia between immediate and delayed induction of labour among late onset mild pre-eclampsia patients. Appendix 1.4. Forest plot showing the difference in eclampsia between immediate and delayed induction of labour among early onset severe pre-eclampsia patients. Appendix 1.5. Forest plot showing the difference in renal failure between immediate and delayed induction of labour among late onset mild pre-eclampsia patients. Appendix 1.6. Forest plot showing the difference in renal failure between immediate and delayed induction of labour among early onset severe pre-eclampsia patients. Appendix 1.7. Forest plot showing the difference in HELLP syndrome between immediate and delayed induction of labour among early onset severe pre-eclampsia patients. Appendix 1.8. Forest plot showing the difference in HELLP syndrome between immediate and delayed induction of labour among late onset mild pre-eclampsia patients. Appendix 1.9. Forest plot showing the difference in thromboembolic disease between immediate and delayed induction of labour among late onset mild pre-eclampsia patients. Appendix 1.10. Forest plot showing the difference in postpartum haemorrhage between immediate and delayed induction of labour among late onset mild pre-eclampsia patients. Appendix 1.11. Forest plot showing the difference in caesarean section between immediate and delayed induction of labour among late onset mild pre-eclampsia patients. Appendix 1.12. Forest plot showing the difference in caesarean section between immediate and delayed induction of labour among early onset mild pre-eclampsia p [file 12884_2020_3407_MOESM2_ESM.zip › Appendix 1.13R5.png]

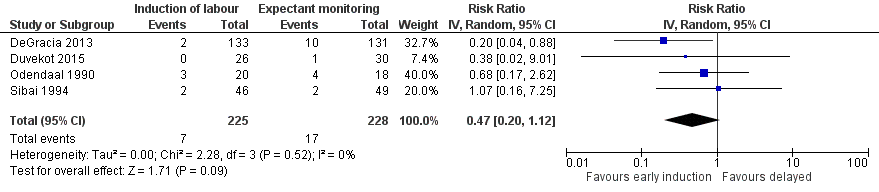

Supplement: Supplementary file 2 — Additional file 2: Appendix 1.1. Forest plot showing the difference in maternal mortality between immediate and delayed induction of labour among early onset severe pre-eclampsia patients. Appendix 1.2. Forest plot showing the difference in maternal mortality between immediate and delayed induction of labour among late onset mild pre-eclampsia patients. Appendix 1.3. Forest plot showing the difference in eclampsia between immediate and delayed induction of labour among late onset mild pre-eclampsia patients. Appendix 1.4. Forest plot showing the difference in eclampsia between immediate and delayed induction of labour among early onset severe pre-eclampsia patients. Appendix 1.5. Forest plot showing the difference in renal failure between immediate and delayed induction of labour among late onset mild pre-eclampsia patients. Appendix 1.6. Forest plot showing the difference in renal failure between immediate and delayed induction of labour among early onset severe pre-eclampsia patients. Appendix 1.7. Forest plot showing the difference in HELLP syndrome between immediate and delayed induction of labour among early onset severe pre-eclampsia patients. Appendix 1.8. Forest plot showing the difference in HELLP syndrome between immediate and delayed induction of labour among late onset mild pre-eclampsia patients. Appendix 1.9. Forest plot showing the difference in thromboembolic disease between immediate and delayed induction of labour among late onset mild pre-eclampsia patients. Appendix 1.10. Forest plot showing the difference in postpartum haemorrhage between immediate and delayed induction of labour among late onset mild pre-eclampsia patients. Appendix 1.11. Forest plot showing the difference in caesarean section between immediate and delayed induction of labour among late onset mild pre-eclampsia patients. Appendix 1.12. Forest plot showing the difference in caesarean section between immediate and delayed induction of labour among early onset mild pre-eclampsia p [file 12884_2020_3407_MOESM2_ESM.zip › Appendix 1.14R5.png]

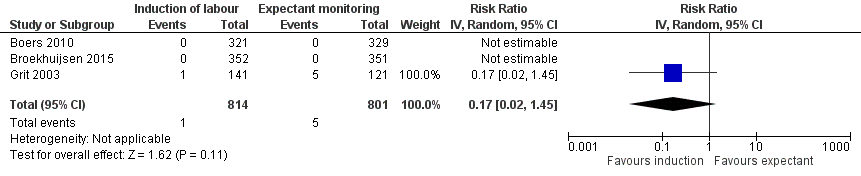

Supplement: Supplementary file 2 — Additional file 2: Appendix 1.1. Forest plot showing the difference in maternal mortality between immediate and delayed induction of labour among early onset severe pre-eclampsia patients. Appendix 1.2. Forest plot showing the difference in maternal mortality between immediate and delayed induction of labour among late onset mild pre-eclampsia patients. Appendix 1.3. Forest plot showing the difference in eclampsia between immediate and delayed induction of labour among late onset mild pre-eclampsia patients. Appendix 1.4. Forest plot showing the difference in eclampsia between immediate and delayed induction of labour among early onset severe pre-eclampsia patients. Appendix 1.5. Forest plot showing the difference in renal failure between immediate and delayed induction of labour among late onset mild pre-eclampsia patients. Appendix 1.6. Forest plot showing the difference in renal failure between immediate and delayed induction of labour among early onset severe pre-eclampsia patients. Appendix 1.7. Forest plot showing the difference in HELLP syndrome between immediate and delayed induction of labour among early onset severe pre-eclampsia patients. Appendix 1.8. Forest plot showing the difference in HELLP syndrome between immediate and delayed induction of labour among late onset mild pre-eclampsia patients. Appendix 1.9. Forest plot showing the difference in thromboembolic disease between immediate and delayed induction of labour among late onset mild pre-eclampsia patients. Appendix 1.10. Forest plot showing the difference in postpartum haemorrhage between immediate and delayed induction of labour among late onset mild pre-eclampsia patients. Appendix 1.11. Forest plot showing the difference in caesarean section between immediate and delayed induction of labour among late onset mild pre-eclampsia patients. Appendix 1.12. Forest plot showing the difference in caesarean section between immediate and delayed induction of labour among early onset mild pre-eclampsia p [file 12884_2020_3407_MOESM2_ESM.zip › Appendix 1.15R5.png]

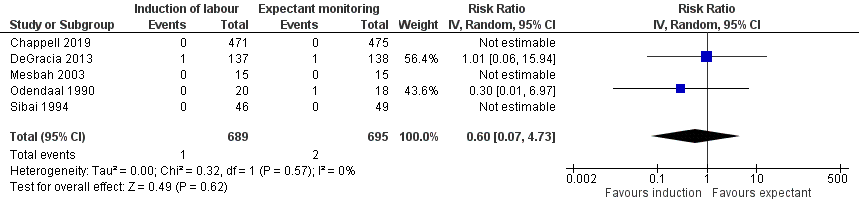

Supplement: Supplementary file 2 — Additional file 2: Appendix 1.1. Forest plot showing the difference in maternal mortality between immediate and delayed induction of labour among early onset severe pre-eclampsia patients. Appendix 1.2. Forest plot showing the difference in maternal mortality between immediate and delayed induction of labour among late onset mild pre-eclampsia patients. Appendix 1.3. Forest plot showing the difference in eclampsia between immediate and delayed induction of labour among late onset mild pre-eclampsia patients. Appendix 1.4. Forest plot showing the difference in eclampsia between immediate and delayed induction of labour among early onset severe pre-eclampsia patients. Appendix 1.5. Forest plot showing the difference in renal failure between immediate and delayed induction of labour among late onset mild pre-eclampsia patients. Appendix 1.6. Forest plot showing the difference in renal failure between immediate and delayed induction of labour among early onset severe pre-eclampsia patients. Appendix 1.7. Forest plot showing the difference in HELLP syndrome between immediate and delayed induction of labour among early onset severe pre-eclampsia patients. Appendix 1.8. Forest plot showing the difference in HELLP syndrome between immediate and delayed induction of labour among late onset mild pre-eclampsia patients. Appendix 1.9. Forest plot showing the difference in thromboembolic disease between immediate and delayed induction of labour among late onset mild pre-eclampsia patients. Appendix 1.10. Forest plot showing the difference in postpartum haemorrhage between immediate and delayed induction of labour among late onset mild pre-eclampsia patients. Appendix 1.11. Forest plot showing the difference in caesarean section between immediate and delayed induction of labour among late onset mild pre-eclampsia patients. Appendix 1.12. Forest plot showing the difference in caesarean section between immediate and delayed induction of labour among early onset mild pre-eclampsia p [file 12884_2020_3407_MOESM2_ESM.zip › Appendix 1.16R5.png]

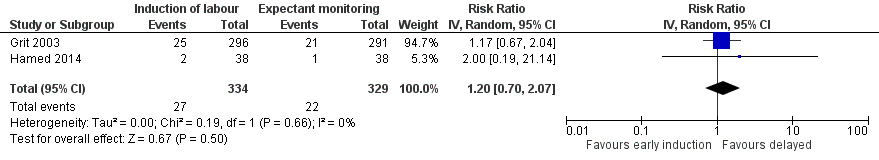

Supplement: Supplementary file 2 — Additional file 2: Appendix 1.1. Forest plot showing the difference in maternal mortality between immediate and delayed induction of labour among early onset severe pre-eclampsia patients. Appendix 1.2. Forest plot showing the difference in maternal mortality between immediate and delayed induction of labour among late onset mild pre-eclampsia patients. Appendix 1.3. Forest plot showing the difference in eclampsia between immediate and delayed induction of labour among late onset mild pre-eclampsia patients. Appendix 1.4. Forest plot showing the difference in eclampsia between immediate and delayed induction of labour among early onset severe pre-eclampsia patients. Appendix 1.5. Forest plot showing the difference in renal failure between immediate and delayed induction of labour among late onset mild pre-eclampsia patients. Appendix 1.6. Forest plot showing the difference in renal failure between immediate and delayed induction of labour among early onset severe pre-eclampsia patients. Appendix 1.7. Forest plot showing the difference in HELLP syndrome between immediate and delayed induction of labour among early onset severe pre-eclampsia patients. Appendix 1.8. Forest plot showing the difference in HELLP syndrome between immediate and delayed induction of labour among late onset mild pre-eclampsia patients. Appendix 1.9. Forest plot showing the difference in thromboembolic disease between immediate and delayed induction of labour among late onset mild pre-eclampsia patients. Appendix 1.10. Forest plot showing the difference in postpartum haemorrhage between immediate and delayed induction of labour among late onset mild pre-eclampsia patients. Appendix 1.11. Forest plot showing the difference in caesarean section between immediate and delayed induction of labour among late onset mild pre-eclampsia patients. Appendix 1.12. Forest plot showing the difference in caesarean section between immediate and delayed induction of labour among early onset mild pre-eclampsia p [file 12884_2020_3407_MOESM2_ESM.zip › Appendix 1.17R5.png]

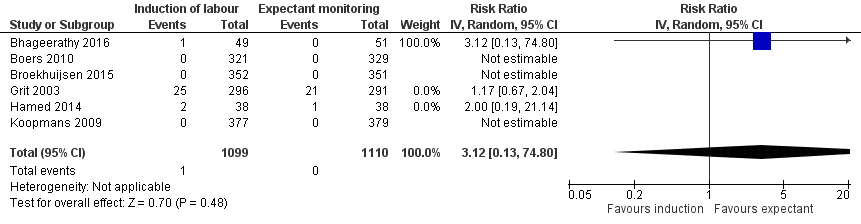

Supplement: Supplementary file 2 — Additional file 2: Appendix 1.1. Forest plot showing the difference in maternal mortality between immediate and delayed induction of labour among early onset severe pre-eclampsia patients. Appendix 1.2. Forest plot showing the difference in maternal mortality between immediate and delayed induction of labour among late onset mild pre-eclampsia patients. Appendix 1.3. Forest plot showing the difference in eclampsia between immediate and delayed induction of labour among late onset mild pre-eclampsia patients. Appendix 1.4. Forest plot showing the difference in eclampsia between immediate and delayed induction of labour among early onset severe pre-eclampsia patients. Appendix 1.5. Forest plot showing the difference in renal failure between immediate and delayed induction of labour among late onset mild pre-eclampsia patients. Appendix 1.6. Forest plot showing the difference in renal failure between immediate and delayed induction of labour among early onset severe pre-eclampsia patients. Appendix 1.7. Forest plot showing the difference in HELLP syndrome between immediate and delayed induction of labour among early onset severe pre-eclampsia patients. Appendix 1.8. Forest plot showing the difference in HELLP syndrome between immediate and delayed induction of labour among late onset mild pre-eclampsia patients. Appendix 1.9. Forest plot showing the difference in thromboembolic disease between immediate and delayed induction of labour among late onset mild pre-eclampsia patients. Appendix 1.10. Forest plot showing the difference in postpartum haemorrhage between immediate and delayed induction of labour among late onset mild pre-eclampsia patients. Appendix 1.11. Forest plot showing the difference in caesarean section between immediate and delayed induction of labour among late onset mild pre-eclampsia patients. Appendix 1.12. Forest plot showing the difference in caesarean section between immediate and delayed induction of labour among early onset mild pre-eclampsia p [file 12884_2020_3407_MOESM2_ESM.zip › Appendix 1.18R5.png]

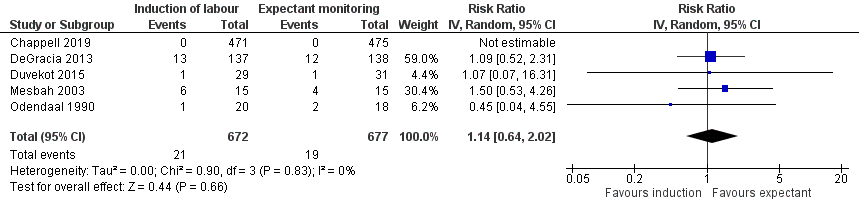

Supplement: Supplementary file 2 — Additional file 2: Appendix 1.1. Forest plot showing the difference in maternal mortality between immediate and delayed induction of labour among early onset severe pre-eclampsia patients. Appendix 1.2. Forest plot showing the difference in maternal mortality between immediate and delayed induction of labour among late onset mild pre-eclampsia patients. Appendix 1.3. Forest plot showing the difference in eclampsia between immediate and delayed induction of labour among late onset mild pre-eclampsia patients. Appendix 1.4. Forest plot showing the difference in eclampsia between immediate and delayed induction of labour among early onset severe pre-eclampsia patients. Appendix 1.5. Forest plot showing the difference in renal failure between immediate and delayed induction of labour among late onset mild pre-eclampsia patients. Appendix 1.6. Forest plot showing the difference in renal failure between immediate and delayed induction of labour among early onset severe pre-eclampsia patients. Appendix 1.7. Forest plot showing the difference in HELLP syndrome between immediate and delayed induction of labour among early onset severe pre-eclampsia patients. Appendix 1.8. Forest plot showing the difference in HELLP syndrome between immediate and delayed induction of labour among late onset mild pre-eclampsia patients. Appendix 1.9. Forest plot showing the difference in thromboembolic disease between immediate and delayed induction of labour among late onset mild pre-eclampsia patients. Appendix 1.10. Forest plot showing the difference in postpartum haemorrhage between immediate and delayed induction of labour among late onset mild pre-eclampsia patients. Appendix 1.11. Forest plot showing the difference in caesarean section between immediate and delayed induction of labour among late onset mild pre-eclampsia patients. Appendix 1.12. Forest plot showing the difference in caesarean section between immediate and delayed induction of labour among early onset mild pre-eclampsia p [file 12884_2020_3407_MOESM2_ESM.zip › Appendix 1.19R5.png]

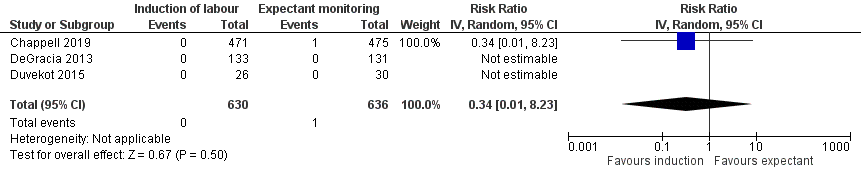

Supplement: Supplementary file 2 — Additional file 2: Appendix 1.1. Forest plot showing the difference in maternal mortality between immediate and delayed induction of labour among early onset severe pre-eclampsia patients. Appendix 1.2. Forest plot showing the difference in maternal mortality between immediate and delayed induction of labour among late onset mild pre-eclampsia patients. Appendix 1.3. Forest plot showing the difference in eclampsia between immediate and delayed induction of labour among late onset mild pre-eclampsia patients. Appendix 1.4. Forest plot showing the difference in eclampsia between immediate and delayed induction of labour among early onset severe pre-eclampsia patients. Appendix 1.5. Forest plot showing the difference in renal failure between immediate and delayed induction of labour among late onset mild pre-eclampsia patients. Appendix 1.6. Forest plot showing the difference in renal failure between immediate and delayed induction of labour among early onset severe pre-eclampsia patients. Appendix 1.7. Forest plot showing the difference in HELLP syndrome between immediate and delayed induction of labour among early onset severe pre-eclampsia patients. Appendix 1.8. Forest plot showing the difference in HELLP syndrome between immediate and delayed induction of labour among late onset mild pre-eclampsia patients. Appendix 1.9. Forest plot showing the difference in thromboembolic disease between immediate and delayed induction of labour among late onset mild pre-eclampsia patients. Appendix 1.10. Forest plot showing the difference in postpartum haemorrhage between immediate and delayed induction of labour among late onset mild pre-eclampsia patients. Appendix 1.11. Forest plot showing the difference in caesarean section between immediate and delayed induction of labour among late onset mild pre-eclampsia patients. Appendix 1.12. Forest plot showing the difference in caesarean section between immediate and delayed induction of labour among early onset mild pre-eclampsia p [file 12884_2020_3407_MOESM2_ESM.zip › Appendix 1.1R5.png]

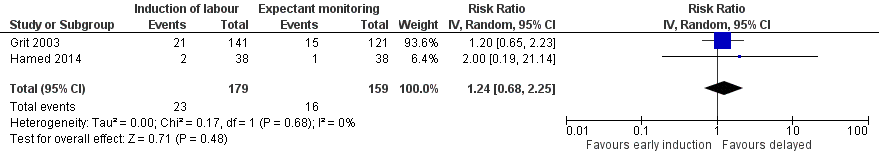

Supplement: Supplementary file 2 — Additional file 2: Appendix 1.1. Forest plot showing the difference in maternal mortality between immediate and delayed induction of labour among early onset severe pre-eclampsia patients. Appendix 1.2. Forest plot showing the difference in maternal mortality between immediate and delayed induction of labour among late onset mild pre-eclampsia patients. Appendix 1.3. Forest plot showing the difference in eclampsia between immediate and delayed induction of labour among late onset mild pre-eclampsia patients. Appendix 1.4. Forest plot showing the difference in eclampsia between immediate and delayed induction of labour among early onset severe pre-eclampsia patients. Appendix 1.5. Forest plot showing the difference in renal failure between immediate and delayed induction of labour among late onset mild pre-eclampsia patients. Appendix 1.6. Forest plot showing the difference in renal failure between immediate and delayed induction of labour among early onset severe pre-eclampsia patients. Appendix 1.7. Forest plot showing the difference in HELLP syndrome between immediate and delayed induction of labour among early onset severe pre-eclampsia patients. Appendix 1.8. Forest plot showing the difference in HELLP syndrome between immediate and delayed induction of labour among late onset mild pre-eclampsia patients. Appendix 1.9. Forest plot showing the difference in thromboembolic disease between immediate and delayed induction of labour among late onset mild pre-eclampsia patients. Appendix 1.10. Forest plot showing the difference in postpartum haemorrhage between immediate and delayed induction of labour among late onset mild pre-eclampsia patients. Appendix 1.11. Forest plot showing the difference in caesarean section between immediate and delayed induction of labour among late onset mild pre-eclampsia patients. Appendix 1.12. Forest plot showing the difference in caesarean section between immediate and delayed induction of labour among early onset mild pre-eclampsia p [file 12884_2020_3407_MOESM2_ESM.zip › Appendix 1.20R5.png]

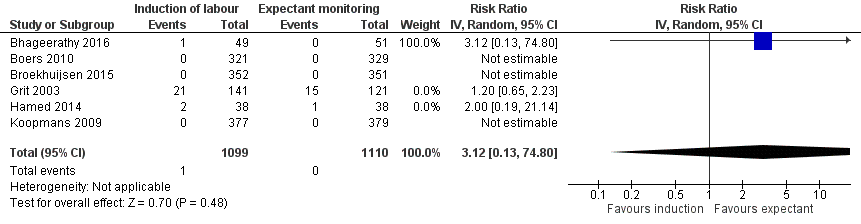

Supplement: Supplementary file 2 — Additional file 2: Appendix 1.1. Forest plot showing the difference in maternal mortality between immediate and delayed induction of labour among early onset severe pre-eclampsia patients. Appendix 1.2. Forest plot showing the difference in maternal mortality between immediate and delayed induction of labour among late onset mild pre-eclampsia patients. Appendix 1.3. Forest plot showing the difference in eclampsia between immediate and delayed induction of labour among late onset mild pre-eclampsia patients. Appendix 1.4. Forest plot showing the difference in eclampsia between immediate and delayed induction of labour among early onset severe pre-eclampsia patients. Appendix 1.5. Forest plot showing the difference in renal failure between immediate and delayed induction of labour among late onset mild pre-eclampsia patients. Appendix 1.6. Forest plot showing the difference in renal failure between immediate and delayed induction of labour among early onset severe pre-eclampsia patients. Appendix 1.7. Forest plot showing the difference in HELLP syndrome between immediate and delayed induction of labour among early onset severe pre-eclampsia patients. Appendix 1.8. Forest plot showing the difference in HELLP syndrome between immediate and delayed induction of labour among late onset mild pre-eclampsia patients. Appendix 1.9. Forest plot showing the difference in thromboembolic disease between immediate and delayed induction of labour among late onset mild pre-eclampsia patients. Appendix 1.10. Forest plot showing the difference in postpartum haemorrhage between immediate and delayed induction of labour among late onset mild pre-eclampsia patients. Appendix 1.11. Forest plot showing the difference in caesarean section between immediate and delayed induction of labour among late onset mild pre-eclampsia patients. Appendix 1.12. Forest plot showing the difference in caesarean section between immediate and delayed induction of labour among early onset mild pre-eclampsia p [file 12884_2020_3407_MOESM2_ESM.zip › Appendix 1.21R5.png]

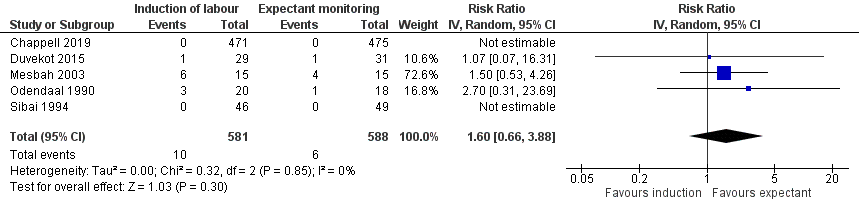

Supplement: Supplementary file 2 — Additional file 2: Appendix 1.1. Forest plot showing the difference in maternal mortality between immediate and delayed induction of labour among early onset severe pre-eclampsia patients. Appendix 1.2. Forest plot showing the difference in maternal mortality between immediate and delayed induction of labour among late onset mild pre-eclampsia patients. Appendix 1.3. Forest plot showing the difference in eclampsia between immediate and delayed induction of labour among late onset mild pre-eclampsia patients. Appendix 1.4. Forest plot showing the difference in eclampsia between immediate and delayed induction of labour among early onset severe pre-eclampsia patients. Appendix 1.5. Forest plot showing the difference in renal failure between immediate and delayed induction of labour among late onset mild pre-eclampsia patients. Appendix 1.6. Forest plot showing the difference in renal failure between immediate and delayed induction of labour among early onset severe pre-eclampsia patients. Appendix 1.7. Forest plot showing the difference in HELLP syndrome between immediate and delayed induction of labour among early onset severe pre-eclampsia patients. Appendix 1.8. Forest plot showing the difference in HELLP syndrome between immediate and delayed induction of labour among late onset mild pre-eclampsia patients. Appendix 1.9. Forest plot showing the difference in thromboembolic disease between immediate and delayed induction of labour among late onset mild pre-eclampsia patients. Appendix 1.10. Forest plot showing the difference in postpartum haemorrhage between immediate and delayed induction of labour among late onset mild pre-eclampsia patients. Appendix 1.11. Forest plot showing the difference in caesarean section between immediate and delayed induction of labour among late onset mild pre-eclampsia patients. Appendix 1.12. Forest plot showing the difference in caesarean section between immediate and delayed induction of labour among early onset mild pre-eclampsia p [file 12884_2020_3407_MOESM2_ESM.zip › Appendix 1.22R5.png]

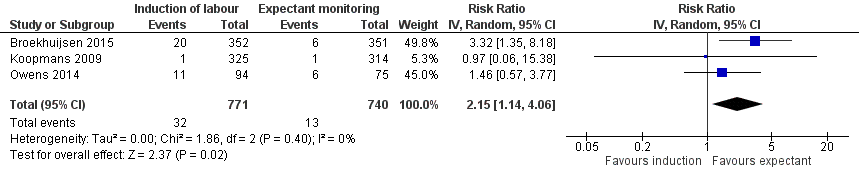

Supplement: Supplementary file 2 — Additional file 2: Appendix 1.1. Forest plot showing the difference in maternal mortality between immediate and delayed induction of labour among early onset severe pre-eclampsia patients. Appendix 1.2. Forest plot showing the difference in maternal mortality between immediate and delayed induction of labour among late onset mild pre-eclampsia patients. Appendix 1.3. Forest plot showing the difference in eclampsia between immediate and delayed induction of labour among late onset mild pre-eclampsia patients. Appendix 1.4. Forest plot showing the difference in eclampsia between immediate and delayed induction of labour among early onset severe pre-eclampsia patients. Appendix 1.5. Forest plot showing the difference in renal failure between immediate and delayed induction of labour among late onset mild pre-eclampsia patients. Appendix 1.6. Forest plot showing the difference in renal failure between immediate and delayed induction of labour among early onset severe pre-eclampsia patients. Appendix 1.7. Forest plot showing the difference in HELLP syndrome between immediate and delayed induction of labour among early onset severe pre-eclampsia patients. Appendix 1.8. Forest plot showing the difference in HELLP syndrome between immediate and delayed induction of labour among late onset mild pre-eclampsia patients. Appendix 1.9. Forest plot showing the difference in thromboembolic disease between immediate and delayed induction of labour among late onset mild pre-eclampsia patients. Appendix 1.10. Forest plot showing the difference in postpartum haemorrhage between immediate and delayed induction of labour among late onset mild pre-eclampsia patients. Appendix 1.11. Forest plot showing the difference in caesarean section between immediate and delayed induction of labour among late onset mild pre-eclampsia patients. Appendix 1.12. Forest plot showing the difference in caesarean section between immediate and delayed induction of labour among early onset mild pre-eclampsia p [file 12884_2020_3407_MOESM2_ESM.zip › Appendix 1.23R5.png]

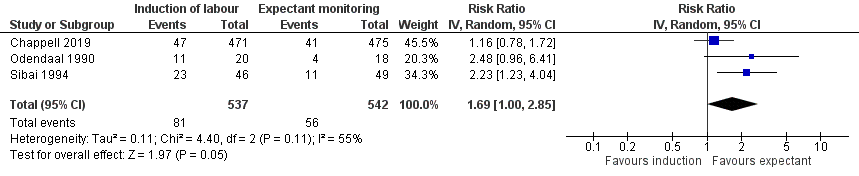

Supplement: Supplementary file 2 — Additional file 2: Appendix 1.1. Forest plot showing the difference in maternal mortality between immediate and delayed induction of labour among early onset severe pre-eclampsia patients. Appendix 1.2. Forest plot showing the difference in maternal mortality between immediate and delayed induction of labour among late onset mild pre-eclampsia patients. Appendix 1.3. Forest plot showing the difference in eclampsia between immediate and delayed induction of labour among late onset mild pre-eclampsia patients. Appendix 1.4. Forest plot showing the difference in eclampsia between immediate and delayed induction of labour among early onset severe pre-eclampsia patients. Appendix 1.5. Forest plot showing the difference in renal failure between immediate and delayed induction of labour among late onset mild pre-eclampsia patients. Appendix 1.6. Forest plot showing the difference in renal failure between immediate and delayed induction of labour among early onset severe pre-eclampsia patients. Appendix 1.7. Forest plot showing the difference in HELLP syndrome between immediate and delayed induction of labour among early onset severe pre-eclampsia patients. Appendix 1.8. Forest plot showing the difference in HELLP syndrome between immediate and delayed induction of labour among late onset mild pre-eclampsia patients. Appendix 1.9. Forest plot showing the difference in thromboembolic disease between immediate and delayed induction of labour among late onset mild pre-eclampsia patients. Appendix 1.10. Forest plot showing the difference in postpartum haemorrhage between immediate and delayed induction of labour among late onset mild pre-eclampsia patients. Appendix 1.11. Forest plot showing the difference in caesarean section between immediate and delayed induction of labour among late onset mild pre-eclampsia patients. Appendix 1.12. Forest plot showing the difference in caesarean section between immediate and delayed induction of labour among early onset mild pre-eclampsia p [file 12884_2020_3407_MOESM2_ESM.zip › Appendix 1.24R5.png]

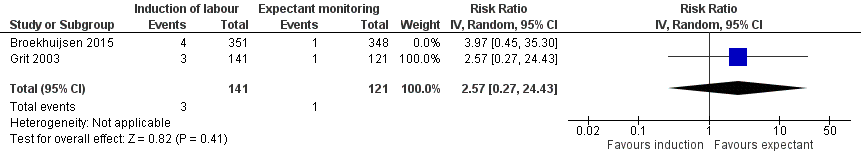

Supplement: Supplementary file 2 — Additional file 2: Appendix 1.1. Forest plot showing the difference in maternal mortality between immediate and delayed induction of labour among early onset severe pre-eclampsia patients. Appendix 1.2. Forest plot showing the difference in maternal mortality between immediate and delayed induction of labour among late onset mild pre-eclampsia patients. Appendix 1.3. Forest plot showing the difference in eclampsia between immediate and delayed induction of labour among late onset mild pre-eclampsia patients. Appendix 1.4. Forest plot showing the difference in eclampsia between immediate and delayed induction of labour among early onset severe pre-eclampsia patients. Appendix 1.5. Forest plot showing the difference in renal failure between immediate and delayed induction of labour among late onset mild pre-eclampsia patients. Appendix 1.6. Forest plot showing the difference in renal failure between immediate and delayed induction of labour among early onset severe pre-eclampsia patients. Appendix 1.7. Forest plot showing the difference in HELLP syndrome between immediate and delayed induction of labour among early onset severe pre-eclampsia patients. Appendix 1.8. Forest plot showing the difference in HELLP syndrome between immediate and delayed induction of labour among late onset mild pre-eclampsia patients. Appendix 1.9. Forest plot showing the difference in thromboembolic disease between immediate and delayed induction of labour among late onset mild pre-eclampsia patients. Appendix 1.10. Forest plot showing the difference in postpartum haemorrhage between immediate and delayed induction of labour among late onset mild pre-eclampsia patients. Appendix 1.11. Forest plot showing the difference in caesarean section between immediate and delayed induction of labour among late onset mild pre-eclampsia patients. Appendix 1.12. Forest plot showing the difference in caesarean section between immediate and delayed induction of labour among early onset mild pre-eclampsia p [file 12884_2020_3407_MOESM2_ESM.zip › Appendix 1.25R5.png]

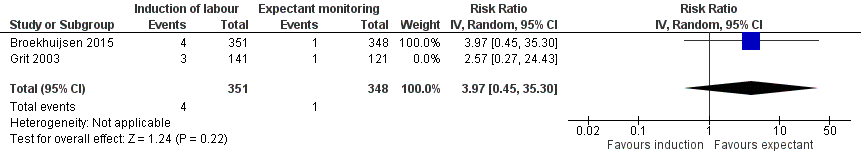

Supplement: Supplementary file 2 — Additional file 2: Appendix 1.1. Forest plot showing the difference in maternal mortality between immediate and delayed induction of labour among early onset severe pre-eclampsia patients. Appendix 1.2. Forest plot showing the difference in maternal mortality between immediate and delayed induction of labour among late onset mild pre-eclampsia patients. Appendix 1.3. Forest plot showing the difference in eclampsia between immediate and delayed induction of labour among late onset mild pre-eclampsia patients. Appendix 1.4. Forest plot showing the difference in eclampsia between immediate and delayed induction of labour among early onset severe pre-eclampsia patients. Appendix 1.5. Forest plot showing the difference in renal failure between immediate and delayed induction of labour among late onset mild pre-eclampsia patients. Appendix 1.6. Forest plot showing the difference in renal failure between immediate and delayed induction of labour among early onset severe pre-eclampsia patients. Appendix 1.7. Forest plot showing the difference in HELLP syndrome between immediate and delayed induction of labour among early onset severe pre-eclampsia patients. Appendix 1.8. Forest plot showing the difference in HELLP syndrome between immediate and delayed induction of labour among late onset mild pre-eclampsia patients. Appendix 1.9. Forest plot showing the difference in thromboembolic disease between immediate and delayed induction of labour among late onset mild pre-eclampsia patients. Appendix 1.10. Forest plot showing the difference in postpartum haemorrhage between immediate and delayed induction of labour among late onset mild pre-eclampsia patients. Appendix 1.11. Forest plot showing the difference in caesarean section between immediate and delayed induction of labour among late onset mild pre-eclampsia patients. Appendix 1.12. Forest plot showing the difference in caesarean section between immediate and delayed induction of labour among early onset mild pre-eclampsia p [file 12884_2020_3407_MOESM2_ESM.zip › Appendix 1.26R5.png]

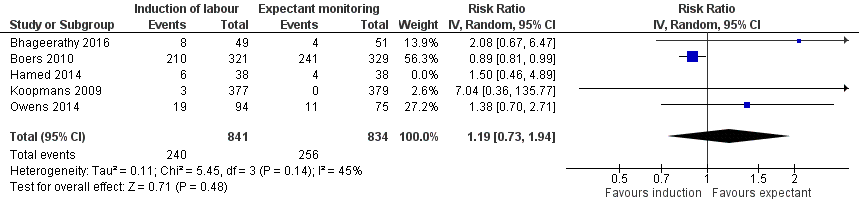

Supplement: Supplementary file 2 — Additional file 2: Appendix 1.1. Forest plot showing the difference in maternal mortality between immediate and delayed induction of labour among early onset severe pre-eclampsia patients. Appendix 1.2. Forest plot showing the difference in maternal mortality between immediate and delayed induction of labour among late onset mild pre-eclampsia patients. Appendix 1.3. Forest plot showing the difference in eclampsia between immediate and delayed induction of labour among late onset mild pre-eclampsia patients. Appendix 1.4. Forest plot showing the difference in eclampsia between immediate and delayed induction of labour among early onset severe pre-eclampsia patients. Appendix 1.5. Forest plot showing the difference in renal failure between immediate and delayed induction of labour among late onset mild pre-eclampsia patients. Appendix 1.6. Forest plot showing the difference in renal failure between immediate and delayed induction of labour among early onset severe pre-eclampsia patients. Appendix 1.7. Forest plot showing the difference in HELLP syndrome between immediate and delayed induction of labour among early onset severe pre-eclampsia patients. Appendix 1.8. Forest plot showing the difference in HELLP syndrome between immediate and delayed induction of labour among late onset mild pre-eclampsia patients. Appendix 1.9. Forest plot showing the difference in thromboembolic disease between immediate and delayed induction of labour among late onset mild pre-eclampsia patients. Appendix 1.10. Forest plot showing the difference in postpartum haemorrhage between immediate and delayed induction of labour among late onset mild pre-eclampsia patients. Appendix 1.11. Forest plot showing the difference in caesarean section between immediate and delayed induction of labour among late onset mild pre-eclampsia patients. Appendix 1.12. Forest plot showing the difference in caesarean section between immediate and delayed induction of labour among early onset mild pre-eclampsia p [file 12884_2020_3407_MOESM2_ESM.zip › Appendix 1.27R5.png]

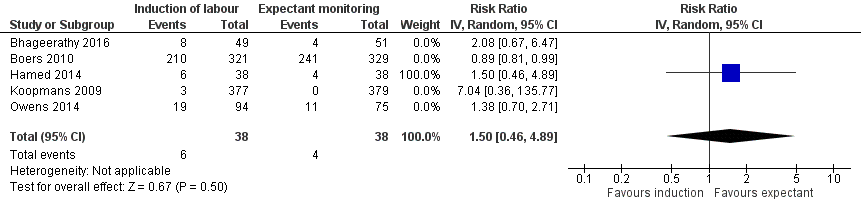

Supplement: Supplementary file 2 — Additional file 2: Appendix 1.1. Forest plot showing the difference in maternal mortality between immediate and delayed induction of labour among early onset severe pre-eclampsia patients. Appendix 1.2. Forest plot showing the difference in maternal mortality between immediate and delayed induction of labour among late onset mild pre-eclampsia patients. Appendix 1.3. Forest plot showing the difference in eclampsia between immediate and delayed induction of labour among late onset mild pre-eclampsia patients. Appendix 1.4. Forest plot showing the difference in eclampsia between immediate and delayed induction of labour among early onset severe pre-eclampsia patients. Appendix 1.5. Forest plot showing the difference in renal failure between immediate and delayed induction of labour among late onset mild pre-eclampsia patients. Appendix 1.6. Forest plot showing the difference in renal failure between immediate and delayed induction of labour among early onset severe pre-eclampsia patients. Appendix 1.7. Forest plot showing the difference in HELLP syndrome between immediate and delayed induction of labour among early onset severe pre-eclampsia patients. Appendix 1.8. Forest plot showing the difference in HELLP syndrome between immediate and delayed induction of labour among late onset mild pre-eclampsia patients. Appendix 1.9. Forest plot showing the difference in thromboembolic disease between immediate and delayed induction of labour among late onset mild pre-eclampsia patients. Appendix 1.10. Forest plot showing the difference in postpartum haemorrhage between immediate and delayed induction of labour among late onset mild pre-eclampsia patients. Appendix 1.11. Forest plot showing the difference in caesarean section between immediate and delayed induction of labour among late onset mild pre-eclampsia patients. Appendix 1.12. Forest plot showing the difference in caesarean section between immediate and delayed induction of labour among early onset mild pre-eclampsia p [file 12884_2020_3407_MOESM2_ESM.zip › Appendix 1.28R5.png]

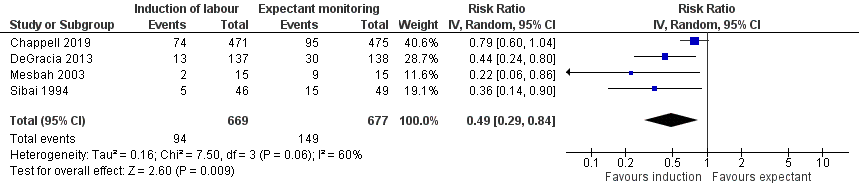

Supplement: Supplementary file 2 — Additional file 2: Appendix 1.1. Forest plot showing the difference in maternal mortality between immediate and delayed induction of labour among early onset severe pre-eclampsia patients. Appendix 1.2. Forest plot showing the difference in maternal mortality between immediate and delayed induction of labour among late onset mild pre-eclampsia patients. Appendix 1.3. Forest plot showing the difference in eclampsia between immediate and delayed induction of labour among late onset mild pre-eclampsia patients. Appendix 1.4. Forest plot showing the difference in eclampsia between immediate and delayed induction of labour among early onset severe pre-eclampsia patients. Appendix 1.5. Forest plot showing the difference in renal failure between immediate and delayed induction of labour among late onset mild pre-eclampsia patients. Appendix 1.6. Forest plot showing the difference in renal failure between immediate and delayed induction of labour among early onset severe pre-eclampsia patients. Appendix 1.7. Forest plot showing the difference in HELLP syndrome between immediate and delayed induction of labour among early onset severe pre-eclampsia patients. Appendix 1.8. Forest plot showing the difference in HELLP syndrome between immediate and delayed induction of labour among late onset mild pre-eclampsia patients. Appendix 1.9. Forest plot showing the difference in thromboembolic disease between immediate and delayed induction of labour among late onset mild pre-eclampsia patients. Appendix 1.10. Forest plot showing the difference in postpartum haemorrhage between immediate and delayed induction of labour among late onset mild pre-eclampsia patients. Appendix 1.11. Forest plot showing the difference in caesarean section between immediate and delayed induction of labour among late onset mild pre-eclampsia patients. Appendix 1.12. Forest plot showing the difference in caesarean section between immediate and delayed induction of labour among early onset mild pre-eclampsia p [file 12884_2020_3407_MOESM2_ESM.zip › Appendix 1.29R5.png]

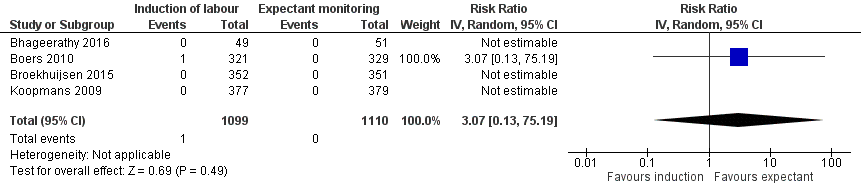

Supplement: Supplementary file 2 — Additional file 2: Appendix 1.1. Forest plot showing the difference in maternal mortality between immediate and delayed induction of labour among early onset severe pre-eclampsia patients. Appendix 1.2. Forest plot showing the difference in maternal mortality between immediate and delayed induction of labour among late onset mild pre-eclampsia patients. Appendix 1.3. Forest plot showing the difference in eclampsia between immediate and delayed induction of labour among late onset mild pre-eclampsia patients. Appendix 1.4. Forest plot showing the difference in eclampsia between immediate and delayed induction of labour among early onset severe pre-eclampsia patients. Appendix 1.5. Forest plot showing the difference in renal failure between immediate and delayed induction of labour among late onset mild pre-eclampsia patients. Appendix 1.6. Forest plot showing the difference in renal failure between immediate and delayed induction of labour among early onset severe pre-eclampsia patients. Appendix 1.7. Forest plot showing the difference in HELLP syndrome between immediate and delayed induction of labour among early onset severe pre-eclampsia patients. Appendix 1.8. Forest plot showing the difference in HELLP syndrome between immediate and delayed induction of labour among late onset mild pre-eclampsia patients. Appendix 1.9. Forest plot showing the difference in thromboembolic disease between immediate and delayed induction of labour among late onset mild pre-eclampsia patients. Appendix 1.10. Forest plot showing the difference in postpartum haemorrhage between immediate and delayed induction of labour among late onset mild pre-eclampsia patients. Appendix 1.11. Forest plot showing the difference in caesarean section between immediate and delayed induction of labour among late onset mild pre-eclampsia patients. Appendix 1.12. Forest plot showing the difference in caesarean section between immediate and delayed induction of labour among early onset mild pre-eclampsia p [file 12884_2020_3407_MOESM2_ESM.zip › Appendix 1.2R5.png]

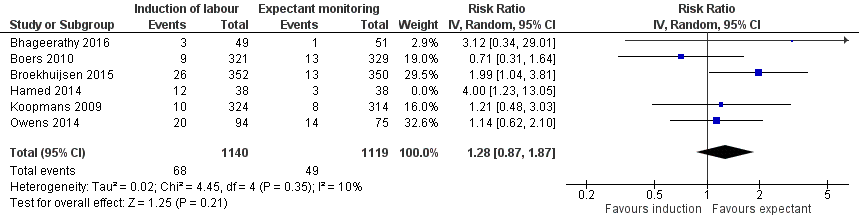

Supplement: Supplementary file 2 — Additional file 2: Appendix 1.1. Forest plot showing the difference in maternal mortality between immediate and delayed induction of labour among early onset severe pre-eclampsia patients. Appendix 1.2. Forest plot showing the difference in maternal mortality between immediate and delayed induction of labour among late onset mild pre-eclampsia patients. Appendix 1.3. Forest plot showing the difference in eclampsia between immediate and delayed induction of labour among late onset mild pre-eclampsia patients. Appendix 1.4. Forest plot showing the difference in eclampsia between immediate and delayed induction of labour among early onset severe pre-eclampsia patients. Appendix 1.5. Forest plot showing the difference in renal failure between immediate and delayed induction of labour among late onset mild pre-eclampsia patients. Appendix 1.6. Forest plot showing the difference in renal failure between immediate and delayed induction of labour among early onset severe pre-eclampsia patients. Appendix 1.7. Forest plot showing the difference in HELLP syndrome between immediate and delayed induction of labour among early onset severe pre-eclampsia patients. Appendix 1.8. Forest plot showing the difference in HELLP syndrome between immediate and delayed induction of labour among late onset mild pre-eclampsia patients. Appendix 1.9. Forest plot showing the difference in thromboembolic disease between immediate and delayed induction of labour among late onset mild pre-eclampsia patients. Appendix 1.10. Forest plot showing the difference in postpartum haemorrhage between immediate and delayed induction of labour among late onset mild pre-eclampsia patients. Appendix 1.11. Forest plot showing the difference in caesarean section between immediate and delayed induction of labour among late onset mild pre-eclampsia patients. Appendix 1.12. Forest plot showing the difference in caesarean section between immediate and delayed induction of labour among early onset mild pre-eclampsia p [file 12884_2020_3407_MOESM2_ESM.zip › Appendix 1.30R5.png]

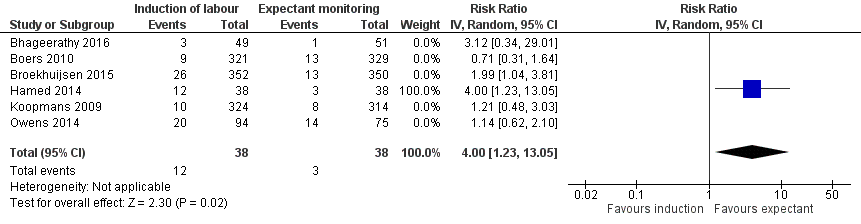

Supplement: Supplementary file 2 — Additional file 2: Appendix 1.1. Forest plot showing the difference in maternal mortality between immediate and delayed induction of labour among early onset severe pre-eclampsia patients. Appendix 1.2. Forest plot showing the difference in maternal mortality between immediate and delayed induction of labour among late onset mild pre-eclampsia patients. Appendix 1.3. Forest plot showing the difference in eclampsia between immediate and delayed induction of labour among late onset mild pre-eclampsia patients. Appendix 1.4. Forest plot showing the difference in eclampsia between immediate and delayed induction of labour among early onset severe pre-eclampsia patients. Appendix 1.5. Forest plot showing the difference in renal failure between immediate and delayed induction of labour among late onset mild pre-eclampsia patients. Appendix 1.6. Forest plot showing the difference in renal failure between immediate and delayed induction of labour among early onset severe pre-eclampsia patients. Appendix 1.7. Forest plot showing the difference in HELLP syndrome between immediate and delayed induction of labour among early onset severe pre-eclampsia patients. Appendix 1.8. Forest plot showing the difference in HELLP syndrome between immediate and delayed induction of labour among late onset mild pre-eclampsia patients. Appendix 1.9. Forest plot showing the difference in thromboembolic disease between immediate and delayed induction of labour among late onset mild pre-eclampsia patients. Appendix 1.10. Forest plot showing the difference in postpartum haemorrhage between immediate and delayed induction of labour among late onset mild pre-eclampsia patients. Appendix 1.11. Forest plot showing the difference in caesarean section between immediate and delayed induction of labour among late onset mild pre-eclampsia patients. Appendix 1.12. Forest plot showing the difference in caesarean section between immediate and delayed induction of labour among early onset mild pre-eclampsia p [file 12884_2020_3407_MOESM2_ESM.zip › Appendix 1.31R5.png]

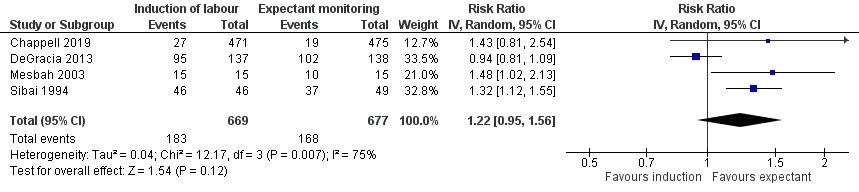

Supplement: Supplementary file 2 — Additional file 2: Appendix 1.1. Forest plot showing the difference in maternal mortality between immediate and delayed induction of labour among early onset severe pre-eclampsia patients. Appendix 1.2. Forest plot showing the difference in maternal mortality between immediate and delayed induction of labour among late onset mild pre-eclampsia patients. Appendix 1.3. Forest plot showing the difference in eclampsia between immediate and delayed induction of labour among late onset mild pre-eclampsia patients. Appendix 1.4. Forest plot showing the difference in eclampsia between immediate and delayed induction of labour among early onset severe pre-eclampsia patients. Appendix 1.5. Forest plot showing the difference in renal failure between immediate and delayed induction of labour among late onset mild pre-eclampsia patients. Appendix 1.6. Forest plot showing the difference in renal failure between immediate and delayed induction of labour among early onset severe pre-eclampsia patients. Appendix 1.7. Forest plot showing the difference in HELLP syndrome between immediate and delayed induction of labour among early onset severe pre-eclampsia patients. Appendix 1.8. Forest plot showing the difference in HELLP syndrome between immediate and delayed induction of labour among late onset mild pre-eclampsia patients. Appendix 1.9. Forest plot showing the difference in thromboembolic disease between immediate and delayed induction of labour among late onset mild pre-eclampsia patients. Appendix 1.10. Forest plot showing the difference in postpartum haemorrhage between immediate and delayed induction of labour among late onset mild pre-eclampsia patients. Appendix 1.11. Forest plot showing the difference in caesarean section between immediate and delayed induction of labour among late onset mild pre-eclampsia patients. Appendix 1.12. Forest plot showing the difference in caesarean section between immediate and delayed induction of labour among early onset mild pre-eclampsia p [file 12884_2020_3407_MOESM2_ESM.zip › Appendix 1.32R5.png]

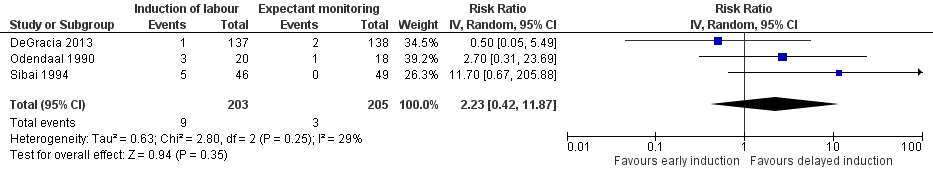

Supplement: Supplementary file 2 — Additional file 2: Appendix 1.1. Forest plot showing the difference in maternal mortality between immediate and delayed induction of labour among early onset severe pre-eclampsia patients. Appendix 1.2. Forest plot showing the difference in maternal mortality between immediate and delayed induction of labour among late onset mild pre-eclampsia patients. Appendix 1.3. Forest plot showing the difference in eclampsia between immediate and delayed induction of labour among late onset mild pre-eclampsia patients. Appendix 1.4. Forest plot showing the difference in eclampsia between immediate and delayed induction of labour among early onset severe pre-eclampsia patients. Appendix 1.5. Forest plot showing the difference in renal failure between immediate and delayed induction of labour among late onset mild pre-eclampsia patients. Appendix 1.6. Forest plot showing the difference in renal failure between immediate and delayed induction of labour among early onset severe pre-eclampsia patients. Appendix 1.7. Forest plot showing the difference in HELLP syndrome between immediate and delayed induction of labour among early onset severe pre-eclampsia patients. Appendix 1.8. Forest plot showing the difference in HELLP syndrome between immediate and delayed induction of labour among late onset mild pre-eclampsia patients. Appendix 1.9. Forest plot showing the difference in thromboembolic disease between immediate and delayed induction of labour among late onset mild pre-eclampsia patients. Appendix 1.10. Forest plot showing the difference in postpartum haemorrhage between immediate and delayed induction of labour among late onset mild pre-eclampsia patients. Appendix 1.11. Forest plot showing the difference in caesarean section between immediate and delayed induction of labour among late onset mild pre-eclampsia patients. Appendix 1.12. Forest plot showing the difference in caesarean section between immediate and delayed induction of labour among early onset mild pre-eclampsia p [file 12884_2020_3407_MOESM2_ESM.zip › Appendix 1.33R5.png]

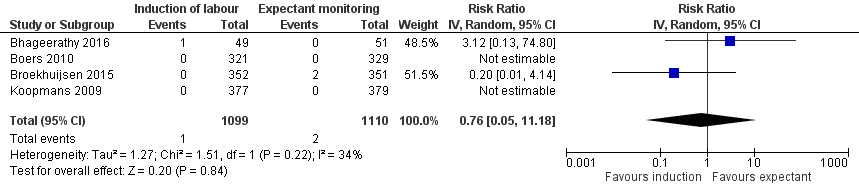

Supplement: Supplementary file 2 — Additional file 2: Appendix 1.1. Forest plot showing the difference in maternal mortality between immediate and delayed induction of labour among early onset severe pre-eclampsia patients. Appendix 1.2. Forest plot showing the difference in maternal mortality between immediate and delayed induction of labour among late onset mild pre-eclampsia patients. Appendix 1.3. Forest plot showing the difference in eclampsia between immediate and delayed induction of labour among late onset mild pre-eclampsia patients. Appendix 1.4. Forest plot showing the difference in eclampsia between immediate and delayed induction of labour among early onset severe pre-eclampsia patients. Appendix 1.5. Forest plot showing the difference in renal failure between immediate and delayed induction of labour among late onset mild pre-eclampsia patients. Appendix 1.6. Forest plot showing the difference in renal failure between immediate and delayed induction of labour among early onset severe pre-eclampsia patients. Appendix 1.7. Forest plot showing the difference in HELLP syndrome between immediate and delayed induction of labour among early onset severe pre-eclampsia patients. Appendix 1.8. Forest plot showing the difference in HELLP syndrome between immediate and delayed induction of labour among late onset mild pre-eclampsia patients. Appendix 1.9. Forest plot showing the difference in thromboembolic disease between immediate and delayed induction of labour among late onset mild pre-eclampsia patients. Appendix 1.10. Forest plot showing the difference in postpartum haemorrhage between immediate and delayed induction of labour among late onset mild pre-eclampsia patients. Appendix 1.11. Forest plot showing the difference in caesarean section between immediate and delayed induction of labour among late onset mild pre-eclampsia patients. Appendix 1.12. Forest plot showing the difference in caesarean section between immediate and delayed induction of labour among early onset mild pre-eclampsia p [file 12884_2020_3407_MOESM2_ESM.zip › Appendix 1.3R5.png]

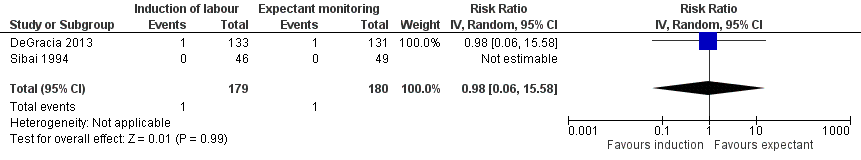

Supplement: Supplementary file 2 — Additional file 2: Appendix 1.1. Forest plot showing the difference in maternal mortality between immediate and delayed induction of labour among early onset severe pre-eclampsia patients. Appendix 1.2. Forest plot showing the difference in maternal mortality between immediate and delayed induction of labour among late onset mild pre-eclampsia patients. Appendix 1.3. Forest plot showing the difference in eclampsia between immediate and delayed induction of labour among late onset mild pre-eclampsia patients. Appendix 1.4. Forest plot showing the difference in eclampsia between immediate and delayed induction of labour among early onset severe pre-eclampsia patients. Appendix 1.5. Forest plot showing the difference in renal failure between immediate and delayed induction of labour among late onset mild pre-eclampsia patients. Appendix 1.6. Forest plot showing the difference in renal failure between immediate and delayed induction of labour among early onset severe pre-eclampsia patients. Appendix 1.7. Forest plot showing the difference in HELLP syndrome between immediate and delayed induction of labour among early onset severe pre-eclampsia patients. Appendix 1.8. Forest plot showing the difference in HELLP syndrome between immediate and delayed induction of labour among late onset mild pre-eclampsia patients. Appendix 1.9. Forest plot showing the difference in thromboembolic disease between immediate and delayed induction of labour among late onset mild pre-eclampsia patients. Appendix 1.10. Forest plot showing the difference in postpartum haemorrhage between immediate and delayed induction of labour among late onset mild pre-eclampsia patients. Appendix 1.11. Forest plot showing the difference in caesarean section between immediate and delayed induction of labour among late onset mild pre-eclampsia patients. Appendix 1.12. Forest plot showing the difference in caesarean section between immediate and delayed induction of labour among early onset mild pre-eclampsia p [file 12884_2020_3407_MOESM2_ESM.zip › Appendix 1.4R5.png]

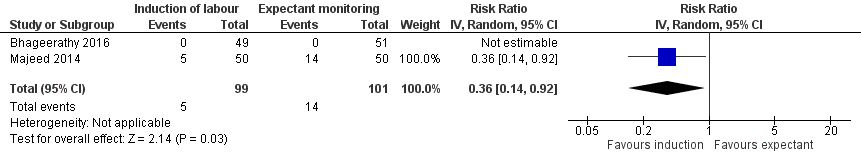

Supplement: Supplementary file 2 — Additional file 2: Appendix 1.1. Forest plot showing the difference in maternal mortality between immediate and delayed induction of labour among early onset severe pre-eclampsia patients. Appendix 1.2. Forest plot showing the difference in maternal mortality between immediate and delayed induction of labour among late onset mild pre-eclampsia patients. Appendix 1.3. Forest plot showing the difference in eclampsia between immediate and delayed induction of labour among late onset mild pre-eclampsia patients. Appendix 1.4. Forest plot showing the difference in eclampsia between immediate and delayed induction of labour among early onset severe pre-eclampsia patients. Appendix 1.5. Forest plot showing the difference in renal failure between immediate and delayed induction of labour among late onset mild pre-eclampsia patients. Appendix 1.6. Forest plot showing the difference in renal failure between immediate and delayed induction of labour among early onset severe pre-eclampsia patients. Appendix 1.7. Forest plot showing the difference in HELLP syndrome between immediate and delayed induction of labour among early onset severe pre-eclampsia patients. Appendix 1.8. Forest plot showing the difference in HELLP syndrome between immediate and delayed induction of labour among late onset mild pre-eclampsia patients. Appendix 1.9. Forest plot showing the difference in thromboembolic disease between immediate and delayed induction of labour among late onset mild pre-eclampsia patients. Appendix 1.10. Forest plot showing the difference in postpartum haemorrhage between immediate and delayed induction of labour among late onset mild pre-eclampsia patients. Appendix 1.11. Forest plot showing the difference in caesarean section between immediate and delayed induction of labour among late onset mild pre-eclampsia patients. Appendix 1.12. Forest plot showing the difference in caesarean section between immediate and delayed induction of labour among early onset mild pre-eclampsia p [file 12884_2020_3407_MOESM2_ESM.zip › Appendix 1.5R5.png]

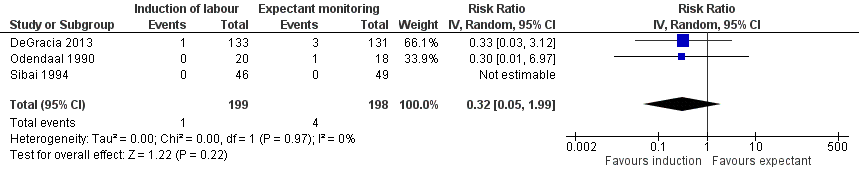

Supplement: Supplementary file 2 — Additional file 2: Appendix 1.1. Forest plot showing the difference in maternal mortality between immediate and delayed induction of labour among early onset severe pre-eclampsia patients. Appendix 1.2. Forest plot showing the difference in maternal mortality between immediate and delayed induction of labour among late onset mild pre-eclampsia patients. Appendix 1.3. Forest plot showing the difference in eclampsia between immediate and delayed induction of labour among late onset mild pre-eclampsia patients. Appendix 1.4. Forest plot showing the difference in eclampsia between immediate and delayed induction of labour among early onset severe pre-eclampsia patients. Appendix 1.5. Forest plot showing the difference in renal failure between immediate and delayed induction of labour among late onset mild pre-eclampsia patients. Appendix 1.6. Forest plot showing the difference in renal failure between immediate and delayed induction of labour among early onset severe pre-eclampsia patients. Appendix 1.7. Forest plot showing the difference in HELLP syndrome between immediate and delayed induction of labour among early onset severe pre-eclampsia patients. Appendix 1.8. Forest plot showing the difference in HELLP syndrome between immediate and delayed induction of labour among late onset mild pre-eclampsia patients. Appendix 1.9. Forest plot showing the difference in thromboembolic disease between immediate and delayed induction of labour among late onset mild pre-eclampsia patients. Appendix 1.10. Forest plot showing the difference in postpartum haemorrhage between immediate and delayed induction of labour among late onset mild pre-eclampsia patients. Appendix 1.11. Forest plot showing the difference in caesarean section between immediate and delayed induction of labour among late onset mild pre-eclampsia patients. Appendix 1.12. Forest plot showing the difference in caesarean section between immediate and delayed induction of labour among early onset mild pre-eclampsia p [file 12884_2020_3407_MOESM2_ESM.zip › Appendix 1.6R5.png]

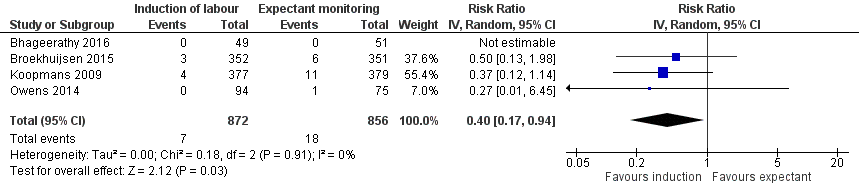

Supplement: Supplementary file 2 — Additional file 2: Appendix 1.1. Forest plot showing the difference in maternal mortality between immediate and delayed induction of labour among early onset severe pre-eclampsia patients. Appendix 1.2. Forest plot showing the difference in maternal mortality between immediate and delayed induction of labour among late onset mild pre-eclampsia patients. Appendix 1.3. Forest plot showing the difference in eclampsia between immediate and delayed induction of labour among late onset mild pre-eclampsia patients. Appendix 1.4. Forest plot showing the difference in eclampsia between immediate and delayed induction of labour among early onset severe pre-eclampsia patients. Appendix 1.5. Forest plot showing the difference in renal failure between immediate and delayed induction of labour among late onset mild pre-eclampsia patients. Appendix 1.6. Forest plot showing the difference in renal failure between immediate and delayed induction of labour among early onset severe pre-eclampsia patients. Appendix 1.7. Forest plot showing the difference in HELLP syndrome between immediate and delayed induction of labour among early onset severe pre-eclampsia patients. Appendix 1.8. Forest plot showing the difference in HELLP syndrome between immediate and delayed induction of labour among late onset mild pre-eclampsia patients. Appendix 1.9. Forest plot showing the difference in thromboembolic disease between immediate and delayed induction of labour among late onset mild pre-eclampsia patients. Appendix 1.10. Forest plot showing the difference in postpartum haemorrhage between immediate and delayed induction of labour among late onset mild pre-eclampsia patients. Appendix 1.11. Forest plot showing the difference in caesarean section between immediate and delayed induction of labour among late onset mild pre-eclampsia patients. Appendix 1.12. Forest plot showing the difference in caesarean section between immediate and delayed induction of labour among early onset mild pre-eclampsia p [file 12884_2020_3407_MOESM2_ESM.zip › Appendix 1.7R5.png]

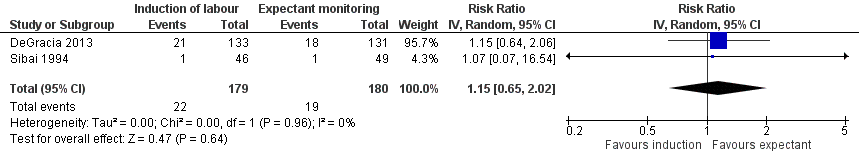

Supplement: Supplementary file 2 — Additional file 2: Appendix 1.1. Forest plot showing the difference in maternal mortality between immediate and delayed induction of labour among early onset severe pre-eclampsia patients. Appendix 1.2. Forest plot showing the difference in maternal mortality between immediate and delayed induction of labour among late onset mild pre-eclampsia patients. Appendix 1.3. Forest plot showing the difference in eclampsia between immediate and delayed induction of labour among late onset mild pre-eclampsia patients. Appendix 1.4. Forest plot showing the difference in eclampsia between immediate and delayed induction of labour among early onset severe pre-eclampsia patients. Appendix 1.5. Forest plot showing the difference in renal failure between immediate and delayed induction of labour among late onset mild pre-eclampsia patients. Appendix 1.6. Forest plot showing the difference in renal failure between immediate and delayed induction of labour among early onset severe pre-eclampsia patients. Appendix 1.7. Forest plot showing the difference in HELLP syndrome between immediate and delayed induction of labour among early onset severe pre-eclampsia patients. Appendix 1.8. Forest plot showing the difference in HELLP syndrome between immediate and delayed induction of labour among late onset mild pre-eclampsia patients. Appendix 1.9. Forest plot showing the difference in thromboembolic disease between immediate and delayed induction of labour among late onset mild pre-eclampsia patients. Appendix 1.10. Forest plot showing the difference in postpartum haemorrhage between immediate and delayed induction of labour among late onset mild pre-eclampsia patients. Appendix 1.11. Forest plot showing the difference in caesarean section between immediate and delayed induction of labour among late onset mild pre-eclampsia patients. Appendix 1.12. Forest plot showing the difference in caesarean section between immediate and delayed induction of labour among early onset mild pre-eclampsia p [file 12884_2020_3407_MOESM2_ESM.zip › Appendix 1.8R5.png]

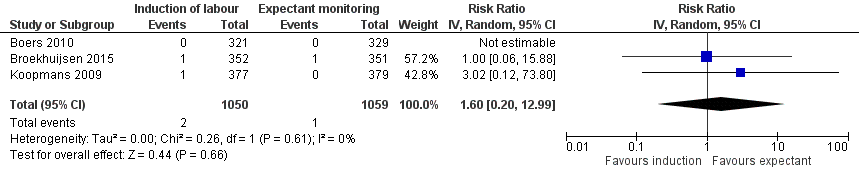

Supplement: Supplementary file 2 — Additional file 2: Appendix 1.1. Forest plot showing the difference in maternal mortality between immediate and delayed induction of labour among early onset severe pre-eclampsia patients. Appendix 1.2. Forest plot showing the difference in maternal mortality between immediate and delayed induction of labour among late onset mild pre-eclampsia patients. Appendix 1.3. Forest plot showing the difference in eclampsia between immediate and delayed induction of labour among late onset mild pre-eclampsia patients. Appendix 1.4. Forest plot showing the difference in eclampsia between immediate and delayed induction of labour among early onset severe pre-eclampsia patients. Appendix 1.5. Forest plot showing the difference in renal failure between immediate and delayed induction of labour among late onset mild pre-eclampsia patients. Appendix 1.6. Forest plot showing the difference in renal failure between immediate and delayed induction of labour among early onset severe pre-eclampsia patients. Appendix 1.7. Forest plot showing the difference in HELLP syndrome between immediate and delayed induction of labour among early onset severe pre-eclampsia patients. Appendix 1.8. Forest plot showing the difference in HELLP syndrome between immediate and delayed induction of labour among late onset mild pre-eclampsia patients. Appendix 1.9. Forest plot showing the difference in thromboembolic disease between immediate and delayed induction of labour among late onset mild pre-eclampsia patients. Appendix 1.10. Forest plot showing the difference in postpartum haemorrhage between immediate and delayed induction of labour among late onset mild pre-eclampsia patients. Appendix 1.11. Forest plot showing the difference in caesarean section between immediate and delayed induction of labour among late onset mild pre-eclampsia patients. Appendix 1.12. Forest plot showing the difference in caesarean section between immediate and delayed induction of labour among early onset mild pre-eclampsia p [file 12884_2020_3407_MOESM2_ESM.zip › Appendix 1.9R5.png]
